# Modulation of Epidermal Transcription Circuits in Psoriasis: New Links between Inflammation and Hyperproliferation

**DOI:** 10.1371/journal.pone.0079253

**Published:** 2013-11-15

**Authors:** William R. Swindell, Andrew Johnston, Xianying Xing, John J. Voorhees, James T. Elder, Johann E. Gudjonsson

**Affiliations:** Department of Dermatology, University of Michigan School of Medicine, Ann Arbor, Michigan, United States of America; University of San Francisco, United States of America

## Abstract

**Background:**

Whole-genome expression profiling has been used to characterize molecular-level differences between psoriasis lesions and normal skin. Pathway analysis, however, is complicated by the fact that expression profiles have been derived from bulk skin biopsies with RNA derived from multiple cell types.

**Results:**

We analyzed gene expression across a large sample of psoriatic (PP) and uninvolved/normal (PN) skin biopsies (*n* = 215 patients). We identified 1975 differentially expressed genes, including 8 associated with psoriasis susceptibility loci. To facilitate pathway analysis, PP versus PN differences in gene expression were analyzed with respect to 235 gene modules, each containing genes with a similar expression pattern in keratinocytes and epidermis. We identified 30 differentially expressed modules (DEMs) biased towards PP-increased or PP-decreased expression. These DEMs were associated with regulatory axes involving cytokines (e.g., IFN-γ, IL-17A, TNF-α), transcription factors (e.g., STAT1, NF-κB, E2F, RUNX1) and chromatin modifiers (SETDB1). We identified an interferon-induced DEM with genes encoding anti-viral proteins (designated “STAT1-57”), which was activated in psoriatic epidermis but repressed following biologic therapy. Genes within this DEM shared a motif near the transcription start site resembling the interferon-stimulated response element (ISRE).

**Conclusions:**

We analyzed a large patient cohort and developed a new approach for delineating epidermis-specific pathways and regulatory mechanisms that underlie altered gene expression in psoriasis. Our findings highlight previously unrecognized “transcription circuits” that can provide targets for development of non-systemic therapies.

## Introduction

Psoriasis is a chronic inflammatory disease characterized by the development of plaque-like skin lesions with adherent silvery scales. The disease affects 2–3% of the population and negatively impacts quality of life and psychological well-being [1]. Cutaneous psoriasis frequently occurs with psoriatic arthritis, and a number of psoriasis co-morbidities have been recognized, including obesity [2], metabolic syndrome [3], hypertension [4], and increased use of anti-depressants [5]. In recent years, application of genomic technologies has provided groundwork for hypothesis-driven investigations of psoriasis disease mechanisms. Genome-wide association studies, for instance, have now identified several dozen psoriasis susceptibility loci [6], and the functional genomic profile of psoriasis lesions has emerged from microarray- and RNAseq-based expression profiling [7]–[15]. Additionally, small noncoding RNAs differentially expressed between psoriasis lesions and normal skin have been identified [16], as well as genomic regions showing altered methylation in lesional skin [17]. These advances have bolstered our understanding of pathogenic mechanisms in psoriasis, yielding an overall picture in which T-cells, antigen-presenting cells and pro-inflammatory cytokines are centrally involved as an initial “trigger” that, ultimately, invokes a broader inflammatory response and abnormal keratinocyte (KC) proliferation [18], [19]. Despite this progress, however, our understanding of pathways altered in psoriasis lesions is far from complete, and we lack efficacious non-systemic treatments that precisely target pathogenic processes within the epidermis [20].

Psoriasis lesion development depends upon interactions among several cell types, but KCs are responsible for thickening of the epidermis (acanthosis) and to a large extent the clinical appearance of plaques. Within lesions, KCs respond to the inflammatory microenvironment by undergoing excessive proliferation and impaired differentiation, as well as by releasing chemokines and cytokines that sustain plaque development [18], [19]. KCs, therefore, have a multifaceted and non-passive role, acting as both responders and effectors in a feed-forward inflammatory reaction [18], [19]. Ultimately, KC activity in psoriasis is governed by stimulation of key membrane receptors, such as the glucocorticoid receptor (GR) [21], G-protein coupled purinergic receptor P2Y (P2RY11) [22] and epidermal growth factor receptor (EGFR) [23]. Receptor stimulation triggers a cascade of signaling events leading to nuclear translocation of transcription factors (TFs) and DNA-binding proteins. Within psoriasis lesions, for instance, the MAPK and ERK1/2 signaling pathways are activated [24], [25]. These and other signaling pathways converge upon a set of TFs that mediate the transcriptional response and eventual synthesis of proteins allowing for hyperproliferation. In particular, NF-κB has emerged as an important TF that can be activated by TNF and other cytokines in psoriasis lesions, potentially providing a mechanism by which KCs integrate inflammatory signals with downstream events allowing for extensive cell division [26]. Aside from NF-κB, however, KC activity in psoriasis also depends crucially on other TFs and pathways, with one example being the p63/ZNF750/KLF4 axis and its effects on terminal KC differentiation [27]–[29].

Genome-wide expression analyses of psoriasis lesions have provided an objective and data-driven approach for identification of disease-associated pathways [7]–[15]. This has led to an accumulation of potentially useful data [30], [31], but interpretation is complicated by the fact that expression has mostly been measured in bulk skin samples, with RNA pools derived from a collection of heterogeneous cell types (e.g., KCs, fibroblasts, T-cells, macrophages and dendritic cells). As a consequence, it is difficult to determine whether genes with altered expression arise from intrinsic modulation of KC-specific pathways, since alternatively, gene expression shifts could be explained by modulation of pathways in other cell types as well [9], [15]. Additionally, some genes may show altered expression in bulk samples of psoriasis lesions because of shifts in cellular composition; for instance, lesions contain more CD4+ T-cells than normal skin and may thus show increased expression of genes expressed specifically by CD4+ T-cells [9], [15]. This complicates pathway analyses based upon sets of genes with altered expression in bulk samples of lesional versus normal skin. Nevertheless, an advantage of bulk tissue analysis is that expression is analyzed in minimally-disrupted cells, without the additional enzymatic and mechanical dissociation steps needed when isolating a specific cell population or tissue layer [32]. Consequently, in psoriasis, and in other contexts, there is a need for analysis protocols that extract signals from the different transcription “circuits” at work in specific cell types, despite the fact that expression is measured in RNA pools derived from a heterogeneous collection of cell types [9], [15].

This study provides a novel analysis of the psoriasis transcriptome based upon a large set of lesional (PP) and non-lesional (PN) biopsies (*n* = 215 patients). This sample set is the largest of any that has previously been analyzed, and was generated by pooling data from four published studies that had used the same full-genome Affymetrix array platform [11], [31], [33], [34]. Given this rich data source, we take steps toward a more definitive characterization of the psoriasis transcriptome, by identifying differentially expressed genes (DEGs) using robust non-parametric statistics, and by drawing connections between these DEGs and susceptibility loci from genome-wide association studies. Within this context, we have developed a strategy for interpreting PP versus PN shifts in gene expression in terms of epidermis-specific pathways and transcription circuits. This was done by first organizing epidermis-expressed genes into distinct co-expression modules based upon the expression pattern of genes across primary KC cultures and epidermal isolates. Modules were then cross-referenced with expression patterns in psoriasis lesions, leading to the identification of differentially expressed modules (DEMs) biased towards PP-increased or PP-decreased expression. DEMs provide a window into the epidermis-driven expression patterns of psoriasis lesions and offer new insights into mechanisms that drive KC hyperproliferation and psoriasis plaque development.

## Results

### Meta-analysis of Gene Expression Identifies 1068 Increased and 907 Decreased Genes in Psoriasis Lesions (*n* = 215 Patients)

We evaluated gene expression in paired lesional (PP) and uninvolved (PN) skin biopsies from 215 patients (Affymetrix Human Genome 2.0 array) [11], [31], [33], [34] (Table S1). Differentially expressed genes (DEGs) were identified using a non-parametric statistical approach (Wilcoxon rank-sum test). We identified 1975 DEGs with significantly altered expression in PP versus PN skin, including 1068 PP-increased DEGs (FDR <0.05 and median FC >1.50) and 907 PP-decreased DEGs (FDR <0.05 and median FC <0.67). Genes increased most in PP skin included *SERPINB4*, *S100A12* and *TCN1*, while genes most strongly decreased included *BTC*, *WIF1* and *THRSP* (Figure S1). While these genes were, on average, the most strongly altered in psoriasis lesions, we also noted substantial variation among the 215 individuals (Figure S1). For each of the top-ranked PP-increased DEGs, for instance, there were in fact some individuals showing *decreased* expression in PP skin (Figure S1). Likewise, for each of the top-ranked PP-decreased DEGs, it was possible to identify individuals for whom expression was *increased* in PP skin (Figure S1). This variation was also present among samples derived from the same source study (Figure S2). We were unable to identify any genes with PP-decreased expression in all 215 patients, and could identify only 3 genes with PP-increased expression in all 215 patients (i.e., *CD274*, FC = 6.77; *SOX7*, FC = 2.08; *INA*, FC = 1.85). These results suggest a near absence of strictly universal expression patterns in psoriatic skin and underscore the importance of large sample sizes within this context.

### Psoriasis DEGs Partially Overlap with Genes Near Susceptibility Loci for Psoriasis and other Autoimmune Diseases (Inflammatory Bowel, Celiac and Crohn’s Disease)

Psoriasis DEGs were further evaluated to assess enrichment for functional annotations and overlap with findings from genome-wide association studies (GWAS). Among the 1068 PP-increased DEGs, significantly overrepresented Gene Ontology (GO) biological process terms were related to cell division/mitosis, immune response, inflammation, and stress or defense response (Figure S3). Among the 907 PP-decreased DEGs, overrepresented GO terms were related to development, cell movement, cell adhesion and differentiation (Figure S4). PP-increased DEGs included a disproportionately high number of genes associated with susceptibility loci for inflammatory bowel disease, celiac disease and Crohn’s disease (Figure S5; P≤4.8×10^−5^; FDR ≤0.007; Fisher’s Exact Test), and we identified 5 PP-increased DEGs associated with all three conditions (*PVT1*, *REL*, *IL18RAP*, *GSDMC*, *PUS10*; Figure S6). To a lesser degree, PP-increased genes overlapped with genes near susceptibility loci for psoriatic arthritis (P = 0.001; FDR = 0.051) and psoriasis (P = 0.004; FDR = 0.093). These trends were absent with respect to PP-decreased DEGs, which were instead associated with susceptibility loci for metabolic syndrome and body size traits (Figure S5). Of 40 genes associated with psoriasis susceptibility loci, expression was usually altered significantly in psoriasis lesions (P<0.001); however, in most cases, the effect size, as measured by median fold-change (PP/PN) was small (Figure S7). Nevertheless, 7 of the PP-increased DEGs were associated with psoriasis susceptibility loci (*LCE3D*, *IFIH1*, *GRHL3*, *IL12B*, *PRSS53*, *REL* and *NOS2*), and 1 PP-decreased DEG was near a psoriasis susceptibility locus (*SDC4*) (Figure S7).

### Identification of 30 Differentially Expressed Modules (DEMs) with Bias Towards Increased or Decreased Expression in Psoriasis Lesions

The altered expression of some genes in psoriasis lesions can be explained by shifts in cellular composition rather than the activation or repression of pathways within epidermal cells [9], [15]. To identify trends related to the modulation of epidermis-specific pathways, 13391 epidermis-expressed genes were clustered based upon their normalized expression across an independent set of 149 microarray samples, where each sample measured expression in primary KCs or epidermal isolates. Based upon the resulting dendrogram, we identified 235 co-expression modules, each of which contained between 25 and 197 genes. We reference each module by citing the best-annotated member gene (i.e., most GO BP terms) and the number of genes belonging to the module; for example, we identified “CDK1-38” as a module containing 38 genes, of which *CDK1* was annotated with the largest number of GO BP terms.

**Figure 1:**
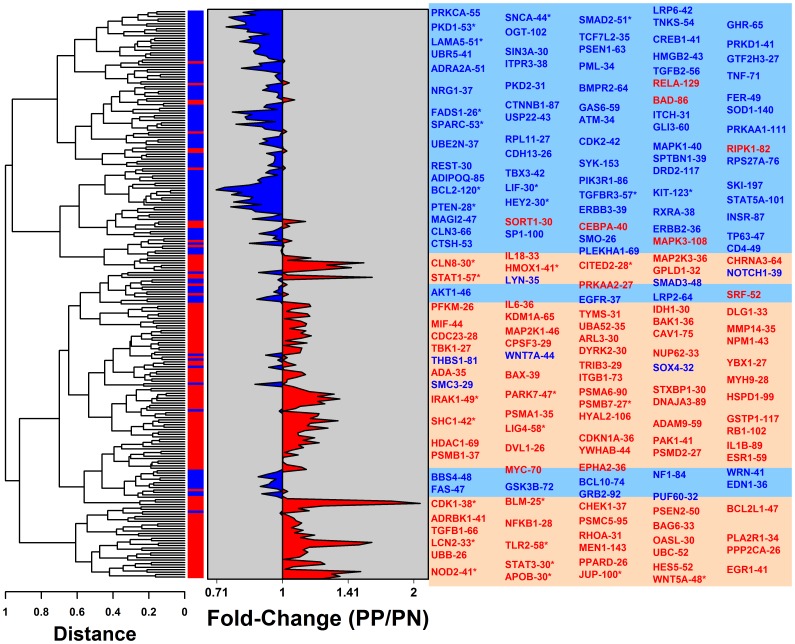
The epidermal gene expression landscape: Regions repressed and activated in psoriasis lesions. We identified 235 gene modules based upon co-expression patterns in KC and epidermis microarray samples. These 235 modules were clustered, with distance between modules proportional to Euclidean distance between module medoids. For each module, a “median of medians” approach was used to calculate a score reflecting the typical PP/PN fold-change among member genes (horizontal axis). Labels for selected modules are listed in the right margin, with modules showing the most PP-increased or PP-decreased bias listed in the left-most columns. Red labels denote PP-increased modules (i.e., median PP/PN fold-change greater than 1) and blue labels denote PP-decreased modules (i.e., median PP/PN fold-change less than 1). Modules with significant bias towards PP-increased or PP-decreased expression are indicated by an asterisk symbol (FDR <0.05 by GSEA with median FC >1.25 or FC <0.80). In the right margin, red shade boxes denote landscape regions with a concentration of PP-increased modules, and blue shade boxes denote regions in which PP-decreased modules are concentrated.

**Figure 2:**
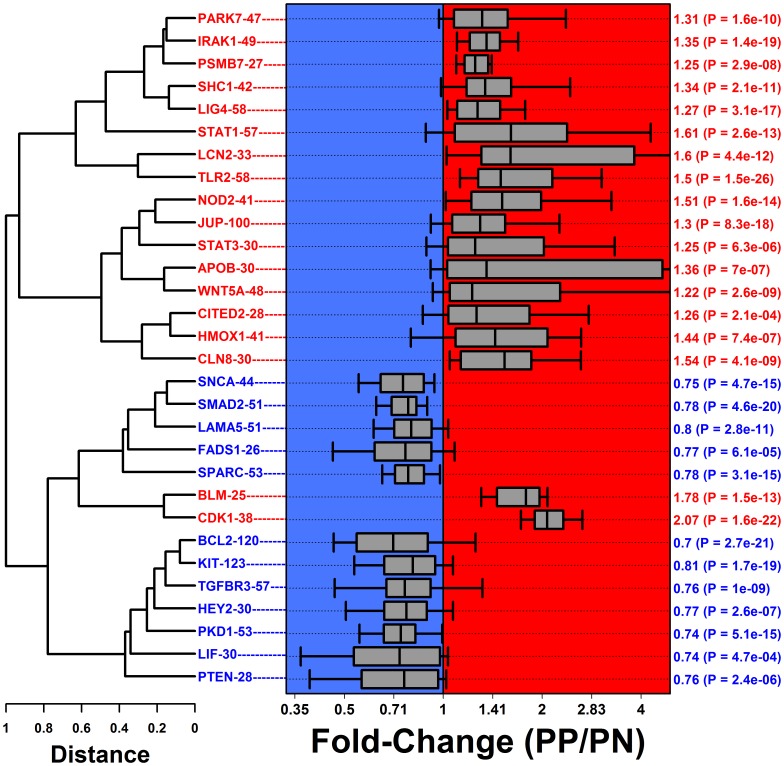
Differentially expressed module (DEM) cluster analysis. We identified 30 modules with bias towards increased or decreased expression in PP skin compared to PN skin. Modules were clustered based upon expression patterns in KC and epidermis microarray samples, with distance between modules proportional to the Euclidean distance between module medoids. Grey boxes span the middle 50% of fold-change estimates (PP/PN) among module genes, and whiskers span the middle 80% of fold-change estimates (PP/PN) among module genes. The right margin lists the median fold-change (PP/PN) among genes belonging to each module, along with a p-value testing whether module genes were disproportionately increased or decreased in psoriasis lesions (FDR <0.05 in each case; GSEA).

The 235 modules were grouped according to similarity of their expression pattern (KCs/epidermis) to provide a high-level view of the epidermis “transcription landscape” (Figure 1). This revealed distinct regional trends, with one region featuring a concentration of PP-increased modules, and another region containing a concentration of PP-decreased modules (Figure 1). Overall, we identified 30 differentially expressed modules (DEMs) with bias towards PP-increased or PP-decreased expression (Table S2 and Figure 2). This included 28 modules for which the median fold-change (PP/PN) of member genes was, relative to all epidermis-expressed genes, significantly biased towards PP-increased or PP-decreased expression (FDR <0.05 by GSEA with median FC >1.25 or median FC <0.80). Additionally, the 30 DEMs include one module with a larger-than-expected proportion of PP-increased DEGs (WNT5A-48), and one module with a significantly large proportion of PP-decreased DEGs (KIT-123) (FDR <0.05; Fisher’s Exact Test; Figure S8).

PP-increased and PP-decreased DEMs exhibited dissimilar expression patterns in KCs and epidermal isolates. This was evident from our initial cluster analysis of DEM expression medoids, since PP-increased DEMs were mostly grouped in branches apart from PP-decreased DEMs (Figure 2). In many cases, moreover, expression medoids of PP-increased DEMs were positively correlated with one another, but negatively correlated with PP-decreased DEMs (Figure S9). Likewise, expression profiles of PP-decreased DEMs were often positively correlated, but negatively correlated with PP-increased DEMs (Figure S9). Finally, visualizing DEM medoids in two-dimensional principal component space revealed a clear trend: PP-increased DEMs and PP-decreased DEMs occupied distinct regions, with the two groups differing significantly with respect to both PC axes (P≤4.1×10^−3^; Figure S10). DEMs trending towards increased and decreased expression in psoriasis lesions thus showed divergent expression patterns across an independent set of KC and epidermis microarray samples.

### Psoriasis DEMs are Functionally Associated with Immunity, Mitosis, Lipids, RNA Processing, Development/Differentiation and Apoptosis

Groups of co-expressed genes will often encode proteins that work together as serial components of a pathway that executes a narrow set of biological functions [35]–[37]. To assess this possibility, we determined whether DEM genes were enriched with respect to annotations from several sources (i.e., Gene Ontology, Reactome, KEGG and PharmGKB databases; Table S2). DEMs could be categorized as predominantly associated with the following core processes: immunity, mitosis, lipids, RNA processing, development/differentiation and apoptosis (Figure S11). In several cases, DEMs were enriched with genes associated with more than one category; for instance, genes within LIG4-58 were associated with both immunity and mitosis (Figure S11). In addition, DEMs contained genes disproportionately associated with T cell differentiation, KC differentiation, NF-κB cascade, leukocyte activation, cell proliferation and tumor necrosis factor (Figure S11). KIT-123, in particular, was a PP-decreased DEM predominantly associated with RNA processing and development/differentiation, but also included genes associated with diverse processes, such as T helper cell differentiation, αβ T cell activation, cell proliferation and mitotic cell cycle (Table S2). DEMs were thus linked to processes known to drive psoriasis plaque development, with some DEMs representing “shared circuits” connecting immune responses to epidermal proliferation and differentiation.

### DEMs have Altered Expression within the Psoriatic Epidermis and are Associated with Differential Methylation

**Figure 3:**
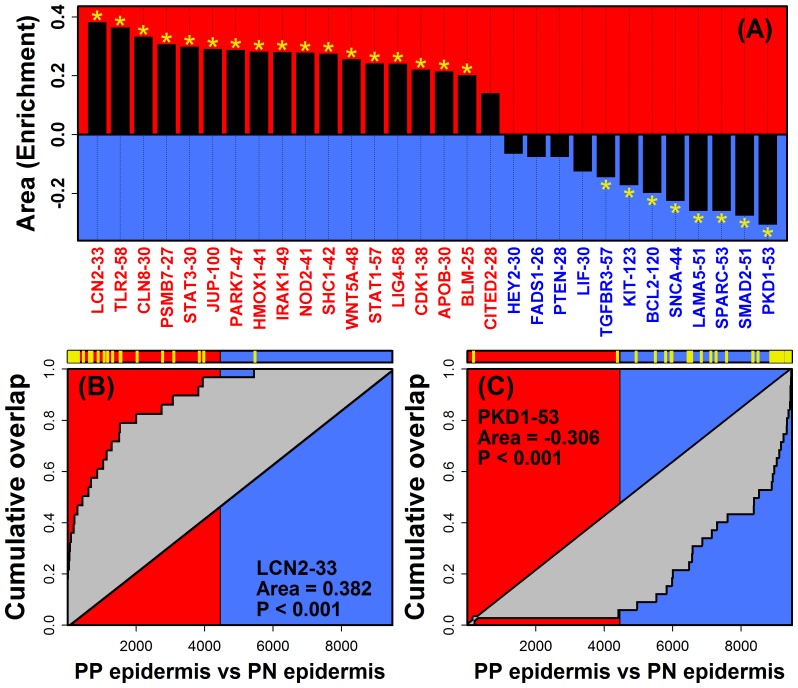
DEMs are biased towards increased or decreased expression in psoriasis epidermis (laser capture microdissection). The 30 DEMs were evaluated to determine if member genes were disproportionately induced or repressed in LCM-dissected PP epidermis (GSEA). (A) GSEA detection rate curve area statistics for each of the 30 DEMs (red labels, PP-increased DEMs; blue labels, PP-decreased DEMs). Significant statistics are denoted by an asterisk symbol (FDR <0.05, yellow asterisk). Parts (B) and (C) show GSEA results for LCN2-33 and PKD1-53, respectively. Human genes were ranked according to their expression difference in LCM-dissected PP epidermis relative to LCM-dissected PN epidermis (horizontal axis). Low ranks were assigned to genes elevated in LCM-dissected PP epidermis (left, red region), while high ranks were assigned to genes repressed in LCM-dissected PP epidermis (right, blue region). Yellow hash marks (top) denote placement of DEM genes with respect to each ranking, and the curve in each figure tracks the cumulative overlap of DEM genes from left to right (vertical axis). Enrichment of DEM genes among genes elevated in LCM-dissected PP epidermis is indicated by a cumulative overlap curve above the diagonal (i.e., positive area statistic; Figure B). Enrichment of DEM genes among genes repressed in LCM-dissected PP epidermis is indicated by a cumulative overlap curve below the diagonal (i.e., negative area statistic; Figure C).

Our central hypothesis is that DEMs correspond to transcription circuits active within the epidermal compartment of psoriasis lesions. We thus evaluated whether DEM genes were also biased towards increased or decreased expression in LCM-dissected PP epidermis as compared to LCM-dissected epidermis from PN skin (Figure 3) [38]. As expected, PP-increased DEMs were in each case elevated in LCM-dissected PP epidermis, while PP-decreased DEMs were repressed in LCM-dissected PP-epidermis (Figure 3). These trends were significant with respect to all but 4 of the 30 DEMs (FDR <0.05; GSEA; Figure 3). The most significant result was observed for TLR2-58 (P = 3.1×10^−18^; Figure 3), which included several genes strongly elevated in LCM-dissected psoriatic epidermis (e.g., *CD24*, *IDH3A*, *NAMPT*, *S100A9*, *RAB38* and *SPRR1A*). Since methylation of genome sites may contribute to DEM regulation, we also investigated whether sites near DEM genes were hyper- or hypo-methylated (Figure S12; *n* = 8 paired PP and PN samples) [17]. We identified one PP-decreased DEM with genes biased towards hyper-methylation in psoriasis lesions (i.e., PTEN-28; P = 0.01; FDR = 0.16; GSEA), along with two PP-increased DEMs with genes biased towards hypo-methylation (i.e., STAT3-30 and WNT5A-48; P≤0.023; FDR ≤0.23; GSEA) (Figure S12).

### Psoriasis DEMs are Associated with Regulatory Axes Connecting Cytokine Signals to Transcription Factors (STAT1, E2F, RUNX1 and NF-κB) and the SETDB1 Histone Methyltransferase

KC proliferation and differentiation are influenced by the unique cytokine environment of psoriasis lesions, which is believed to direct activation of intracellular cascades and key transcription factors [39]. We therefore assessed whether DEM genes were cytokine-responsive, based upon expression responses observed in a set of 42 experiments in which epidermal cells had been treated with cytokines. Nearly all experiments were *in vitro* studies of cultured KCs or reconstituted epidermis (see Methods). DEMs were also assessed to determine if sequences near member genes were enriched for motifs recognized by transcription factors and other DNA-binding proteins. This was done for each DEM by screening a dictionary of 1937 motifs, which we assembled by pooling experimentally-derived motif collections from public and private databases (see Methods).

**Figure 4:**
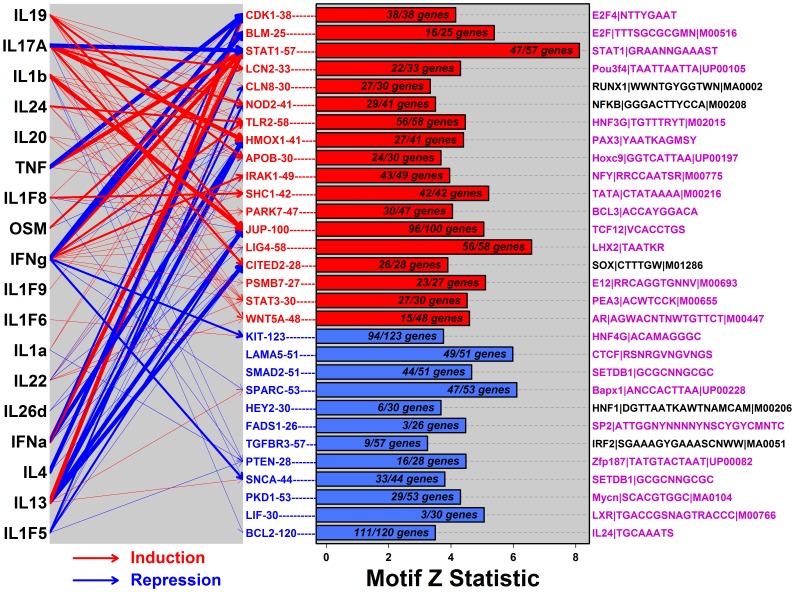
Psoriasis DEMs are associated with regulatory axes connecting cytokine signals to transcription factors (STAT1, E2F, RUNX1 and NF-κB) and the SETDB1 histone methyltransferase. The 30 DEMs were evaluated to assess whether member genes were regulated in cytokine-treated KCs (left) and to identify transcription factor binding sites enriched in sequences adjacent to genes within each DEM (right). Left: Cytokine-DEM relationships were investigated based upon transcriptional responses observed in KCs following treatment with one of the 18 listed cytokines. Red arrows indicate that DEM genes were disproportionately increased by cytokine treatment (P<10^−3^ by both GSEA and Fisher’s exact test). Blue arrows indicate that DEM genes were disproportionately decreased by cytokine treatment (P<10^−3^ by both GSEA and Fisher’s exact test). Arrow thickness corresponds to the strength of the cytokine-DEM association (thin arrows, P<10^−3^; middle thickness, P<10^−6^; thickest arrows, P<10^−9^). Right: DEMs were evaluated to identify the single motif most highly enriched in non-coding intergenic regions adjacent to member genes. For each DEM, the motif showing the highest enrichment is listed in the right margin, with significant motifs shown in magenta font (FDR <0.10). The *Z* statistic quantifies the degree of motif enrichment, based upon a comparison of motif frequency in intergenic regions near DEM genes versus frequency in all other intergenic regions associated with epidermis-expressed genes (semiparametric generalized additive logistic model; see Methods). Within each bar, the text indicates the fraction of DEM genes potentially regulated by the motif listed in the right margin, i.e., the fraction of DEM genes for which at least one site matching the indicated motif was identified within flanking intergenic sequence.

Consistent with cytokine-driven expression in psoriasis epidermis, PP-increased DEMs frequently included cytokine-regulated genes, with several PP-increased DEMs containing genes induced or repressed by multiple cytokines (Figure 4). Cytokine-regulation of PP-decreased DEMs, in contrast, was weaker, although not altogether absent (Figure 4). Among the PP-increased DEMs, several emerged as “cytokine hubs” with genes responsive to multiple cytokines (e.g., STAT1-57, STAT3-30, TLR2-58, JUP-100 and WNT5A-48; Figure 4). Genes belonging to STAT1-57, for example, were induced by six cytokines (IL-1α, IL-1F9, IFN-α, IFN-γ, TNF and OSM) and repressed by two others (IL-4 and IL-17A) (P<0.001; GSEA and Fisher’s exact test; Figure 4). Among PP-decreased DEMs, only PTEN-28 was a strong cytokine hub, with genes repressed by four cytokines (i.e., IL-1α, IL-1F5, IL-1F8 and IL-17A (P<0.001; GSEA and Fisher’s exact test; Figure 4). Individual cytokines varied from predominant DEM-inducers (e.g., IL-19, IL-17A and IL-1β) to predominant DEM-repressors (e.g., IL-1F5, IL-13 and IL-4), with certain cytokines showing a balance between inductive and repressive effects (e.g., IFN-γ) (Figure 4).

Transcription responses downstream of cytokine stimulation are mediated by the activation or repression of transcription factors and DNA-binding proteins [40], [41]. For 25 of the 30 DEMs, we identified a motif significantly enriched with respect to non-coding intergenic sequences adjacent to DEM genes (FDR<0.10 with motif present within an adjacent sequence for at least 5% of DEM genes). Overall, the strongest evidence was obtained with respect to STAT1-57, for which we identified enrichment of STAT1 binding sites among adjacent sequences (FDR = 9.0×10^−13^; Figure 4; discussed further below). Otherwise, we obtained evidence to support E2F, RUNX1, NF-κB and SETDB1 as candidate mediators of altered expression in psoriatic epidermis. CDK1-38 and BLM-25, for instance, each contained genes adjacent to sequences enriched with E2F recognition sites (Figure 4). Since both CDK1-38 and BLM-25 were neighbors in the epidermal transcription landscape (Figure 1), and since both contained genes important for mitosis and cell cycle control (Figure S11), this result is consistent with prior work demonstrating regulation of the cell cycle by E2F in epidermis [42]. Genes within other PP-increased DEMs were located near sequences enriched with NF-κB or RUNX1 binding sites (i.e., NOD2-41 and CLN8-30). NOD2-41, for instance, is a PP-increased DEM including genes associated with epidermis development (e.g., *GRHL3*, *IVL* and *OVOL1*; Table S2), and sequences adjacent to these genes were most enriched for motifs recognized by NF-κB (P = 4.6×10^−4^; FDR = 0.30), with at least one NF-κB motif identified for 29 of the 41 DEM genes (Figure 4). Similarly, CLN8-30 is a PP-increased DEM containing genes involved in development and cellular response to TNF (e.g., *PYDC1* and *ZFAND6*; Table S2), and nearly all genes within this DEM (27 of 30) were located near sequences with a RUNX1 binding site (P = 9.5×10^−5^; FDR = 0.09; Figure 4).

Transcriptional silencing through epigenetic chromatin modification is another mechanism by which gene expression can be altered within epidermis [43]. Along these lines, two PP-decreased DEMs, SMAD2-51 and SNCA-44, included genes adjacent to non-coding regions enriched with recognition sites for the histone methyltransferase enzyme SETDB1 (FDR ≤0.032; Figure 4). Both DEMs were neighbors in the transcriptional landscape (Figure 1) and each contained genes associated with RNA metabolism (Table S2). SMAD2-51, moreover, was enriched with genes associated with gene silencing and the organization or remodeling of chromatin (e.g., *EIF2C4*, *SMAD2*, *EP400*, *PHF15*, *PHF2*, *RBBP4* and *RERE*; Table S2). This result highlights the potential role of chromatin modifiers such as SETDB1 as mediators of transcriptional repression within the psoriatic epidermis.

### STAT1-57 is an Interferon-induced DEM Activated in Psoriatic Epidermis but Repressed Following Biologic Therapy (Etanercept, Ixekizumab and Efalizumab)

STAT1-57 emerged as a strong cytokine hub that included mostly PP-increased genes induced *in vitro* by IL-1α, IL-1F9, IFN-α, IFN-γ, TNF and OSM (Figures 4 and S13).

**Figure 5:**
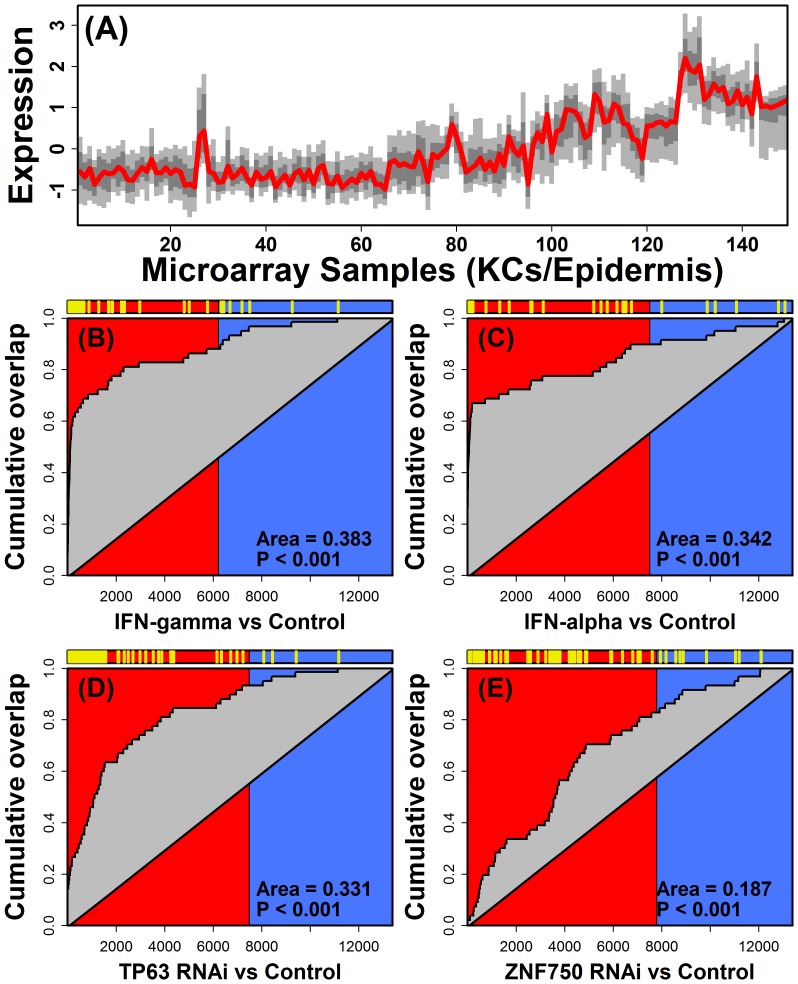
STAT1-57 is an interferon-regulated DEM repressed by the TP63/ZNF750/KLF4 differentiation axis. Part (A) shows the expression profile of the 57 genes belonging to STAT1-57 (KC and epidermis microarray samples). The red line represents median expression among the 57 genes, dark grey outlines expression for the middle 50% of genes, and light grey outlines expression for the middle 80% of genes. Figures (B) – (E) show GSEA results evaluating whether the 57 genes are disproportionately increased or decreased in (B) KCs treated with IFN-γ versus untreated control KCs (GSE2737) (C) KCs treated with IFN-α versus untreated control KCs (GSE36287), (D) KCs with siRNA knockdown of *TP63* versus scambled siRNA (GSE33495) or (E) KCs with siRNA knockdown of *ZNF750* versus scambled siRNA (GSE32685). In each figure (B – E), genes were first ranked according to how their expression is altered in the indicated comparison (horizontal label). Red background denotes genes increased in each comparison while blue background denotes genes decreased in each comparison. Yellow hash marks (top) denote placement of the STAT1-57 genes with respect to each ranking, and the curve in each figure tracks the cumulative overlap of STAT1-57 genes with top-ranked genes from left to right (vertical axis). Enrichment of STAT1-57 members among genes increased in each comparison is indicated by a cumulative overlap curve above the diagonal (i.e., positive area; Figures B – E).

STAT1-57 also included DEAD (Asp-Glu-Ala-Asp) box polypeptide 58 (*DDX58*), which is located near a chromosome 9 psoriasis susceptibility locus [6]. Among STAT1-57 genes, there was strong overrepresentation of genes involved in response to virus (e.g., *DDX58*, *DDX60*, *EIF2AK2*, *HERC5*, *IFI35*, *IFI44*, *IFI44L*, *IFIT1*, *IFITM1*, *IRF9*, *ISG15*, *MX1*, *MX2*, *OAS2*, *OAS3*, *PLSCR1*, *STAT1* and *TRIM22*; P = 8.4×10^−16^). Many STAT1-57 genes were also annotated with the GO BP terms “response to type I interferon” or “response to interferon-gamma” (e.g., *IFI27*, *IFI35*, *IFI6*, *IRF9*, *OAS2* and *OAS3*), and consistent with this, STAT1-57 genes were disproportionately induced in cultured KCs following treatment with either IFN-α or IFN-γ (Figures 4, 5B and 5C; P≤4.9×10^−19^ by GSEA). However, STAT1-57 genes were also disproportionately elevated in hyperproliferative conditions, such as within wound margins (P = 8.8×10^−14^; GSEA) and non-melanoma skin cancers, e.g., squamous cell carcinoma (P = 1.5×10^−10^), basal cell carcinoma (P = 8.5×10^−6^; GSEA) and actinic keratosis (P = 1.2×10^−15^; GSEA). In addition, STAT1-57 genes were repressed in psoriasis lesions following treatment of patients with biologic agents, including anti-TNF therapy (P = 2.1×10^−8^; GSEA; Figure S14A), anti-IL-17A therapy (P = 9.9×10^−8^; GSEA; Figure S14B) and efalizumab (P = 4.5×10^−12^; GSEA; Figure S14C). These effects on STAT1-57 may be due to the immunosuppressant and anti-inflammatory effects of these treatments, since STAT1-57 genes were repressed in KC cultures treated directly with corticosteroid (i.e., dexamethasone; P = 7.8×10^−13^ by GSEA; Figure S14D). There was also a striking trend in which STAT1-57 genes were elevated in KCs following siRNA knockdown of TP63 (P = 5.9×10^−18^; GSEA; Figure 5D), the transcription factor ZNF750 (P = 1.0×10^−6^; GSEA; Figure 5E) and the transcription factor KLF4 (P = 1.3×10^−4^; GSEA). Roughly half of the STAT1-57 genes were in fact elevated following RNAi knockdown of each of these three targets (i.e., p63, ZNF750 and KLF4; e.g., *DDX58*, *IRF9*, *OAS2*, *OAS3* and *STAT1*; P<0.05 for each RNAi treatment by moderated t-test).

### STAT1-57 Genes are Embedded in Genome Regions with Increased Density of an ISRE-like Motif Recognized by Multiple Transcription Factors (STAT1, IRF1 and the ISGF3 Complex)

**Figure 6:**
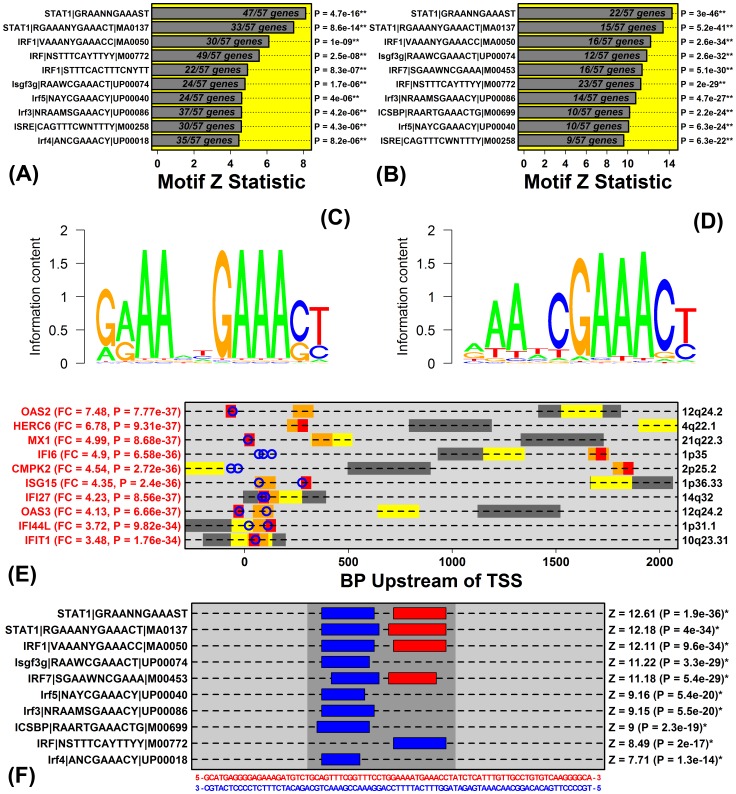
STAT1-57 genes are embedded in genome regions with increased density of an ISRE-like motif recognized by multiple transcription factors (STAT1, IRF1 and the ISGF3 complex). (A) Ten motifs most enriched in non-coding intergenic sequences adjacent to STAT1-57 genes. (B) Ten motifs most enriched in conserved regions 1 KB upstream of STAT1-57 genes. (C) Sequence logo for STAT1|GRAANNGAAAST. (D) Sequence logo for Isgf3g|RAAWCGAAACT|UP00074. (E) Potential regulatory “hotspots” in 2 KB upstream regions upstream of the ten STAT1-57 genes most strongly elevated in psoriasis lesions. Upstream regions were scanned to identify loci with matches to motifs highly enriched with respect to the complete set of STAT1-57 genes (dark grey = best 400 BP window; yellow = best 200 BP window; orange = best 100 BP window; red = best 50 BP window). Blue circles correspond to STAT1|GRAANNGAAAST binding sites. (F) Zoomed view of potential regulatory hotspot 50-130 BP upstream of the *IFI27* transcription start site (hg19, chr14, 94576948–94577028).

We identified a STAT1 recognition site (GRAANNGAAAST) as the most strongly enriched with respect to intergenic regions adjacent to STAT1-57 genes, with at least one such motif identified in sequences adjacent to 47 of the 57 DEM genes (FDR = 9.1×10^−13^; Figure 6A). We repeated these analyses with respect to TSS-proximal regions and noted that enrichment of the GRAANNGAAAST motif strengthened as increasingly narrowed TSS-proximal regions were examined (i.e., intergenic, P = 4.7×10^−16^; 5 KB upstream, P = 9.5×10^−29^; 2 KB upstream, P = 1.9×10^−36^; 1 KB upstream, P = 6.9×10^−44^). Enrichment of the GRAANNGAAAST motif strengthened still further when we considered only the more conserved regions 1 KB upstream of the TSS (P = 3.0×10^−46^; Figure 6B).

The top-ranked GRAANNGAAAST site was recently discovered from an ENCODE experiment in which K562 cells had been treated with IFN-α for 6 hours prior to Chip-seq analysis with an anti-STAT1α antibody [44]. We evaluated TSS-proximal sequences associated with the ten STAT1-57 genes most strongly elevated in PP skin (e.g., *OAS2*, *HERC6*, *MX1*, etc.), and for 9 of these 10 genes there was at least one occurrence of the top-ranked site (i.e., GRAANNGAAAST; see Figure 6E). In each case, moreover, the site was located near the TSS (Figure 6E). With respect to *IFI27*, a gene overexpressed in epithelial cancers [45], we identified a potential transcription “hotspot” immediately upstream of the TSS, which featured two neighboring sites matching the GRAANNGAAAST motif, in addition to several other motifs significantly enriched among sequences upstream of STAT1-57 genes (hg19, chr14, 94576948–94577028; Figure 6F).

**Figure 7:**
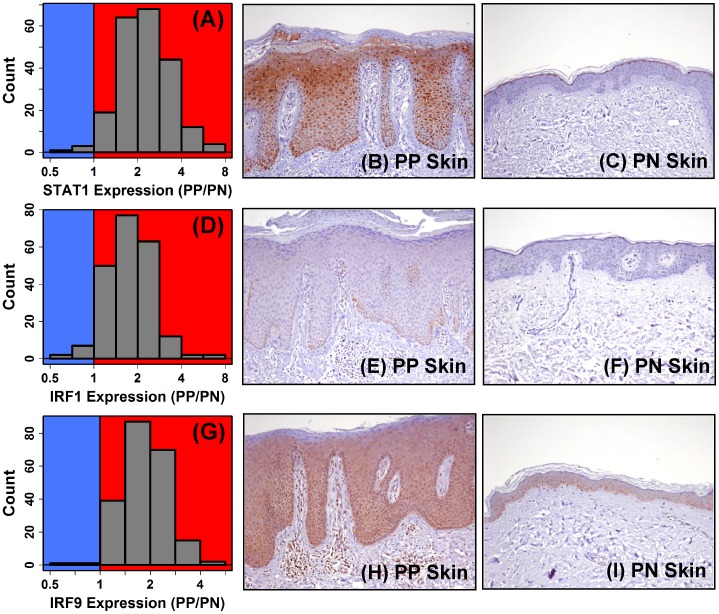
*STAT1* and *IRF9* mRNA are elevated in psoriasis lesions with increased abundance of pSTAT1(ser727) and IRF9 protein in the psoriatic epidermis. Figures (A), (D) and (G) show the PP/PN fold-change distributions for *STAT1*, *IRF1* and *IRF9*, respectively (*n* = 215 patients). Figures (B), (E) and (H) show staining for pSTAT1(ser727), IRF1 and IRF9 in lesional (PP) skin, respectively. Figures (C), (F) and (I) show staining for pSTAT1(ser727), IRF1 and IRF9 in uninvolved (PN) skin, respectively.

STAT1 interacts with DNA as a homodimer or as part of heterotrimeric complex consisting of STAT1, STAT2 and IRF9, i.e., interferon-stimulated gene factor 3 (ISGF3) [46]. In each analysis we performed, motifs recognized by interferon regulatory factors (IRFs) or the ISGF3 complex were secondarily enriched, suggesting that either STAT1 homodimers, the ISGF3 complex, or potentially other IRF proteins (e.g., IRF1) may bind a similar sequence to regulate expression of STAT1-57 genes (Figures 6A and 6B). Most of the top-ranked motifs were in fact similar with respect to their recognition sequence, often with sequence logos resembling the interferon-stimulated response element (ISRE), which is recognized by the ISGF3 complex (e.g., compare Figures 6C and 6D). mRNAs encoding TFs associated with these motifs, moreover, were elevated in PP versus PN skin, including *STAT1* (FC = 2.13; P = 1.3×10^−36^; *n* = 215; Figure 7A), *IRF1* (FC = 1.78; P = 1.8×10^−35^; Figure 7D) and *IRF9* (FC = 1.88; P = 9.9×10^−37^; Figure 7G). However, IHC stains revealed strongly increased abundance of pSTAT1 (ser727) and IRF9 within PP epidermis (Figures 7B, 7C, 7H and 7I), but comparatively weak IRF1 staining (Figures 7E and 7F). Combined with the fact that both *STAT1* and *IRF9* were members of the STAT1-57 gene module, implying their co-expression in KCs/epidermis, these observations support a mechanism by which STAT1 and IRF9 (i.e., ISGF3) cooperatively bind an ISRE-like motif (GRAANNGAAAST) to drive expression of a cytokine hub transcription circuit within the psoriatic epidermis.

## Discussion

**Figure 8:**
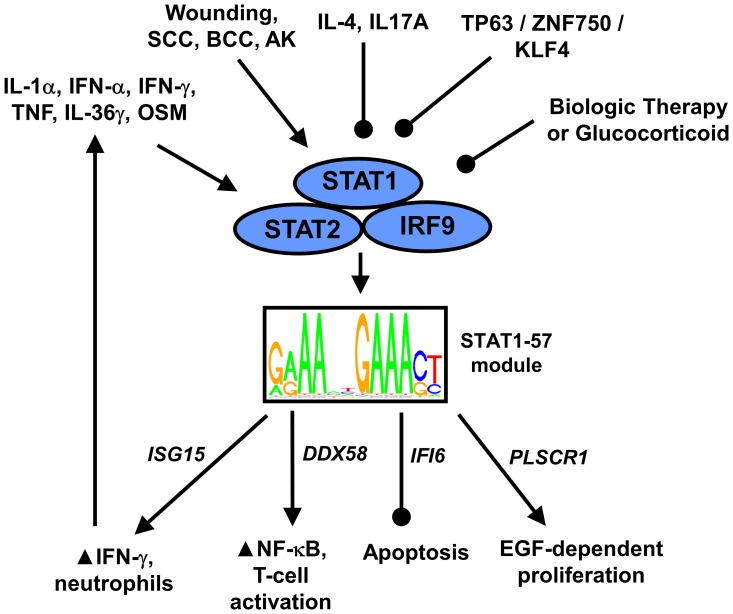
Proposed model for STAT1-57-driven activation of inflammatory and hyperproliferative responses in psoriasis lesions. STAT1-57 is a cytokine hub module consisting of 57 genes co-expressed in KCs and epidermal isolates. In cultured KCs, the 57 genes are biased towards increased expression following treatment with IL-1α, IFN-α, IFN-γ, TNF, IL-36γ and OSM. Similarly, expression of STAT1-57 genes is elevated in hyperproliferative states, such as during wounding and in squamous cell carcinoma (SCC), basal cell carcinoma (BCC) and actinic keratosis (AK). In contrast, in cultured KCs, the 57 genes are biased towards decreased expression following stimulation by IL-17A, IL-4, or glucocorticoid (dexamethasone). RNAi experiments using cultured KCs were also consistent with repression of STAT1-57 genes by the TP63/ZNF750/KLF4 differentiation axis. These upstream signals influence STAT1-57 transcription, potentially by modulating interactions between an ISRE-like motif (GRAANNGAAAST) and the IGSF3 complex (STAT1, STAT2 and IRF9). Activation of STAT1-57 leads to upregulation of mRNAs encoding proteins that promote inflammation (*ISG15* and *DDX58*), inhibit apoptosis (*IFI6*), or which enable complete activation of EGF-dependent signaling (*PLSCR1*). Arrows represent activation and round-tipped lines denote inhibition.

The accumulation of gene expression data from psoriasis lesions has, in recent years, allowed us to characterize the psoriasis transcriptome with unprecedented precision. Bulk skin biopsies of psoriasis lesions, however, are complex because they are composed of a mixture of cell types, including myeloid cells, fibroblasts, and KCs. This complexity has hindered our ability to dissect out the individual “transcription circuits” activated or repressed for any one cell type. To better understand gene expression in psoriasis, therefore, we developed a new strategy for *de novo* detection of epidermal transcription modules, and have here applied this approach to identify differentially expressed modules (DEMs) in lesional skin. We have highlighted one DEM, designated “STAT1-57”, which is induced by multiple cytokines and activated in psoriasis epidermis, as well as certain skin cancers, but is repressed with biologic therapies (i.e., etanercept, efaluzimab and ixekizumab). STAT1-57 includes genes that promote inflammation and NF-κB activation, in combination with genes associated with proliferation and the inhibition of apoptosis (Figure 8). At the level of individual transcription circuits, therefore, our findings reveal a new link between KC immune and proliferative responses, consistent with the idea that KCs are “hardwired” to initiate pro-inflammatory responses concurrent with an expression program that favors proliferation. This is a structural feature of epidermal transcription circuitry with implications for psoriasis disease mechanisms, since it suggests that KC proliferation will proceed in lockstep with development of the inflammation response, ultimately providing a feedforward signal that drives further infiltration of activated immune cells (Figure 8).

STAT1-57 is a “cytokine hub” with genes that, in cultured KCs, are induced by at least six cytokines (IFN-γ, IFN-α, TNF, IL-1α, IL-36γ and OSM) and repressed by two others (IL-17A and IL-4). Within psoriasis lesions, IFN-γ may play a dominant role, since induction of STAT1-57 genes was strongest in KCs following treatment with IFN-γ, with strong, but slightly weaker, induction following treatment with IFN-α (Figure 5). We therefore consider STAT1-57 as primarily a type II IFN-responsive co-expression module, which is, to a lesser degree, also sensitive to type I IFN and other cytokines. These effects of IFN-α and IFN-α on STAT1-57 expression may indeed overwhelm effects of other cytokines, particularly those tending to repress STAT1-57 expression (e.g., IL-17A; Figure 8). Such regulation of STAT1-57 by IFNs suggests potential for positive feedback regulation, since STAT1-57 genes encode proteins that are themselves associated with positive regulation of immune responses (Figure 8). One example is *ISG15*, which encodes a secreted cytokine-like protein that also functions intracellularly as a ubiquitin-like modifier [47]. In psoriasis lesions, *ISG15* mRNA is elevated 4-fold and prior work has confirmed that ISG15 protein is widely distributed in the psoriatic dermis and epidermis [34]. ISG15 has several functions that are not fully understood, but increased ISG15 in psoriasis lesions may amplify or sustain inflammation responses in two ways. First, ISG15 has mitogenic effects on some lymphocyte populations, such as NK-cells, and ISG15 can induce secretion of IFN-γ from T-cells [48], [49]. By stimulating release of IFN-γ from T-cells, ISG15 could contribute to a positive feedback cycle in which the STAT1-57 module, once initially triggered, would become self-sustaining with pathogenic effects (Figure 8). A second important activity of ISG15 is its ability to function as a neutrophil chemoattractant [50]. In psoriasis lesions, neutrophils accumulate and reinforce cytokine cascades by releasing IL-1α, IFN-α and TNF [51]. Since these cytokines, similar to IFN-γ, can stimulate induction of the STAT1-57 gene expression program in KCs, the neutrophil chemoattractant property of ISG15 suggests another mechanism by which STAT1-57 activation may become self-sustaining and chronic (Figure 8).

A second avenue by which STAT1-57 activation can augment inflammatory responses is by heightening the sensitivity of KCs to pathogen-associated molecular patterns (PAMPs) such as virus-derived RNA molecules. STAT1-57, for instance, includes a recently identified psoriasis susceptibility gene *DDX58* [6], which encodes a cytoplasmic RNA helicase known as retinoic acid inducible gene I (*RIG-I*). We found that *DDX58*/*RIG-I* mRNA was elevated 2-fold in psoriasis lesions, and prior work has confirmed that RIG-I protein is more abundant within the epidermal compartment in psoriatic plaques [52], [53]. Under normal conditions, RIG-I is an intracellular sensor that can recognize features of virus-derived dsRNA, such as a 5`-triphosphorylated sequences and polyuridine tracts with interspersed C nucleotides [54]. This primes cells for further activation of defense responses, such that abnormally high abundance of RIG-I sensors may lower the response-threshold for activation of the RIG-I-like receptor (RLR) signaling pathway. Activation of this pathway triggers a signaling cascade mediated by the adaptor protein mitochondrial antiviral signaling protein (MAVS), which in turn has pro-inflammatory effects, including activation of the TFs NF-κB and IRF3/7 [55], and the production of cytokines and chemokines such as IFN-β, IL-1β, IL-6, IL-8, IL-28, IL-29 and RANTES [56]. Apart from the canonical RLR signaling pathway, RIG-I can interact with host RNA to positively regulate NF-κB activity through a post-transcriptional mechanism [57]. In particular, RIG-I binds multiple sites within the 3′-UTR of the *NFKB1* mRNA and this interaction facilitates recruitment of ribosomal proteins to ensure *NFKB1* translation [57]. Finally, a role for RIG-I in T-cell activation is supported by one study of *RIG-I*(−/−) mice, which showed that *RIG-I*(−/−) mice develop a colitis-like phenotype with decreased numbers of CD44^low^CD62L^high^ naïve T-cells, but increased CD44^high^CD62L^low^ effector T cells and CD44^high^CD62L^high^ memory T cells [58].

Characteristic features of KCs within the psoriatic epidermis are hyperproliferation and failure to execute the terminal differentiation program [18], [19]. Such responses must be coupled to the underlying inflammatory and cytokine network, given that systemic therapies directed against cytokines (e.g., IL-17A and TNF) or T-cells have proven efficacious for many patients. Along these lines, STAT1-57 genes were induced in skin biopsies of conditions characterized by heightened proliferation, such as within wound margins, squamous cell carcinoma, basal cell carcinoma and actinic keratosis (Figure 8). For such conditions, STAT1-57 activation may facilitate hyperproliferation through its pro-inflammatory effects, indirectly driving proliferation by helping to shape a pathogenic microenvironment with over-stimulation of cytokine- and chemokine-associated pathways. Alternatively, or in addition, such effects of STAT1-57 may be direct, and mediated by genes encoding proteins that interfere with apoptosis (*IFI6*) or facilitate epidermal growth factor (EGF)-dependent signaling (*PLSCR1*) (Figure 8). *IFI6* encodes interferon alpha-inducible protein 6 (also known as G1P3), which has emerged as a pro-survival anti-apoptotic protein based upon studies using cancer cell lines [59], [60]. In our large cohort, expression of *IFI6* in psoriasis lesions was up-regulated nearly 5-fold, although prior work using RT-PCR and epidermal fractions have estimated 400-fold elevation of *IFI6* in lesional skin [61]. Within the psoriatic epidermis, IFI6 protein is increased, particularly within the extended spinous layer [61]. *IFI6* knockdown by RNA interference in human KCs, moreover, increased apoptosis markers, and further analyses showed that *IFI6* expression was highest in proliferating KCs but decreased during differentiation [61]. These anti-apoptotic effects may synergize with the positive influence of *PLSCR1* on EGF-dependent KC proliferation (Figure 8). *PLSCR1* encodes the phospholipid scramblase 1 plasma membrane protein, which participates in lipid organization and the translocation of phospholipids across the plasma membrane, but is also involved in cell signaling and in some circumstances localizes to the nucleus where it can directly regulate transcription [62]. *PLSCR1* is overexpressed in various cancer cells and interference with *PLSCR1* expression inhibits proliferation, invasion capacity and tumorigenesis [63], [64]. Findings from two studies have delineated a role for *PLSCR1* in EGF-dependent signal transduction [65], [66]. In the first, EGF-stimulation was shown to increase *PLSCR1* expression, PLSCR1 phosphorylation, and association between PLSCR1 and EGF receptor [65]. In the second, it was demonstrated that EGF-stimulated c-Src kinase activity is reduced in *PLSCR1*-deficient cells, suggesting the *PLSCR1* is required for complete activation of the EGF signaling pathway [66].

Many genes have now been identified as differentially expressed in psoriasis lesions, but it has seldom been possible to pinpoint the *cis*-regulatory mechanisms underlying such shifts in gene expression. We identified a motif resembling the interferon-stimulated response element (ISRE) enriched in genome regions near STAT1-57 genes (i.e., G(A/G)AANNGAAA(C/G)T) (Figure 6), and most of the STAT1-57 genes strongly increased in psoriasis lesions featured this motif near the TSS (Figure 6E). The identification of ISRE-like elements among STAT1-57 genes is somewhat surprising, since STAT1-57 genes were more strongly induced by IFN-γ relative to IFN-α (Figure 5). However, IFN-γ activates Jak1/2 and leads to the formation of the STAT1 homodimer, which subsequently recognizes the gamma interferon activation site (GAS) (i.e., TTCN(2-4)GAA) [67], [68]. In contrast, IFN-α/β activates Jak1 and Tyk2 and leads to the formation of the complex IFN-stimulated gene factor 3 (ISGF3), which then binds to ISREs (i.e., GAAA(A)NNGAAA) [67], [69]. At a glance, therefore, enrichment of ISRE-like elements near STAT1-57 genes seems more characteristic of a transcription module directed by type I IFN, rather than type II IFN. We propose, however, that the ISRE-like element we identified is engaged downstream of stimulation by multiple cytokines rather than type I IFN alone. STAT1-57 genes, in particular, were activated by both type I and II IFN, in addition to four other cytokines (i.e., IL-1α, TNF, IL-36γ and OSM; Figure 8). The presence of ISRE-like elements near the TSS of STAT1-57 genes points to multiple TFs or TF complexes as *trans*-regulators, including STAT1, IRF-1, IRF-2 and/or ISGF3. Given that ISGF3 has affinity for ISRE-like elements [67], [69], we favor a model in which STAT1-57 gene expression is activated by the ISGF3 complex in psoriasis lesions (Figure 8), particularly since both *STAT1* and *IRF9* were included among the STAT1-57 genes, and because we and others have demonstrated elevation of pSTAT1(ser727) and IRF9 protein in psoriatic epidermis (Figure 7) [70], [71].

Gene expression in eukaryotic systems appears modular and mechanisms of transcriptional regulation are such that genes may be activated or repressed in concert with one another [72]-[74]. Coordinate regulation of gene expression may indeed be an essential mechanism by which cells orchestrate the transcription of genes encoding components of the same protein complex or proteins participating in the same signaling pathway [35]–[37]. Previous studies of genome-wide expression in psoriasis have commonly focused on differential expression using methods that consider individual genes in isolation. In this study, we have taken an alternative approach, and have emphasized analysis of differentially expressed modules (DEMs) as opposed to differentially expression genes (DEGs). Although KCs within psoriasis lesions exhibit a complex phenotype, characterized by excessive proliferation, failure to reach terminal differentiation, and the release of pro-inflammatory cytokines and chemokines, our findings provide encouraging evidence that such diverse processes may be driven largely by just a small number of hubs within the epidermal transcription circuitry. In future work, we anticipate that similar hubs can be identified with respect to other cell types prominent in psoriasis lesions, such as T-cells, dendritic cells, macrophages and fibroblasts. This should lead to a more complete model of the pathogenic mechanisms at work in psoriasis, and may pave the way towards development of non-systemic therapies that selectively target groups of genes co-regulated within specific cell types.

## Methods

### Ethics Statement

The skin biopsy samples analyzed in this study were obtained in accordance with Declaration of Helsinki principles [11], [31], [33], [34]. Informed written consent was provided by human subjects under protocols approved by Research Review Board Inc. (Richmond Hill, Ontario, Canada), Chesapeake Research Review Inc. (Columbia, MD), and the institutional review boards associated with the University of Michigan (Ann Arbor, MI; IRB no. HUM00037994), Rockefeller University (New York, NY, IRB no. AMA-0674), Royal Adelaide Hospital (Adelaide, Australia) and the Alfred Hospital (Melbourne, Australia).

### Microarray Analysis of Gene Expression in Psoriasis Lesions

Genes with altered expression in psoriasis lesions were identified based upon microarray data from four published studies [11], [31], [33], [34] (Table S1). All data were generated using the same commercial oligonucleotide platform (Affymetrix Human Genome U133 Plus 2.0 Array). This is a comprehensive platform including 54765 probe set features designed to hybridize with transcripts derived from 20184 unique human genes. We excluded from consideration studies of psoriasis lesions in which expression was profiled using platforms with less comprehensive genome coverage [12], [13], [38], [75] (Checklist S1 and Figure S15). Such studies were excluded since the advantage gained by increased sample size would likely have been offset by the introduction of platform-specific heterogeneity into our analysis [31]. For included studies, raw CEL files were obtained from Gene Expression Omnibus (series accessions: GSE13355, GSE14905, GSE30999, GSE34248, GSE41662 and GSE41663). Quality control metrics were calculated for each CEL file, including average background, scale intensity factor, percentage of probe sets called present, RNA degradation score, and statistics derived from the fitting of probe-level models, i.e., Normalized Unscaled Standard Error (NUSE) and Relative Log Expression (RLE) median and interquartile range (IQR) values [76], [77]. Cluster analyses were also performed to identify outlying observations or mislabeled samples. The initial dataset included paired lesional (PP) and non-lesional (PN) biopsies from 219 patients. With respect to 4 patients, quality control analyses indicated that either the PP or PN sample was of poor quality (i.e., GEO sample accessions GSM337287, GSM372350, GSM768062 and GSM1021277). These individuals were excluded and thus our analyses are based upon paired PP and PN samples from 215 patients.

Gene expression scores were calculated for all PP and PN samples using the robust multichip average (RMA) algorithm [78]. Separate normalizations were performed for samples associated with each of the six studies, respectively (Table S1). Although two studies within our analysis did include samples from multiple batches (i.e., GSE13355 and GSE30999), we did not adjust RMA expression values to account for batch-to-batch variation, since in all cases our analysis considers PP versus PN expression differences calculated within individual studies, within batches, and within patients. With this approach, each PP sample is compared only to its respective PN control sample, which was collected at the same office visit and by the same clinician. This approach nullified any impact of batch-to-batch variation in our analysis, which might otherwise impact analyses in which comparisons are made between two independent and unpaired sample groups (e.g., comparisons between lesional/uninvolved skin from patients with normal skin from healthy controls).

The Affymetrix U133 Plus 2.0 array platform includes probe sets designed to target 20184 human genes, but some genes are targeted by multiple “sibling” probe sets [79]. To limit redundancy in the analysis, therefore, a single representative probe set was selected *a priori* for each of the 20184 human genes. In choosing this representative, we preferentially selected a probe set expected to hybridize specifically with transcripts derived from a single gene, i.e., excluding those probe sets with a “_s” or “_x” suffix in the Affymetrix probe set identifier. In some cases, there were multiple probe sets available for a given gene with no expected difference in hybridization specificity. In such cases, the chosen representative was whichever probe set was most highly expressed, on average, across the 430 PP and PN bulk skin biopsy samples included in our analysis.

Following probe set selection, the 20184 genes were filtered to include only those expressed significantly above background in at least 10% of the 430 PP and PN bulk skin biopsy samples. This yielded a final set of 16352 skin-expressed genes, and for each gene, we calculated the PP versus PN expression difference for each of the 215 patients. A Wilcoxon rank sum test was then used to determine if the median PP – PN difference among the 215 patients differed significantly from zero. This test was used since, given the large sample size (*n* = 215), there was no rationale for statistics with shrinkage-based variance estimators as typically utilized in contexts with lower sample size [80], and nevertheless, there was ample power to detect differential expression even with a non-parametric test. To control the false discovery rate (FDR) among tests performed for each of the 16352 human genes, raw p-values derived from the Wilcoxon rank sum test were adjusted using the Benjamini-Hochberg method [81]. These criteria initially yielded 1079 and 917 PP-increased and PP-decreased DEGs, respectively (FDR <0.05 and FC >1.5 or FC <0.67). DEGs were further filtered to include only those for which median FC was greater than 1 with respect to each of the six studies individually (PP-increased DEGs) or less than 1 with respect to each of the six studies individually (PP-decreased DEGs). This yielded the final set of 1068 and 907 PP-increased and PP-decreased DEGs, respectively.

### Comparison of Psoriasis DEGs to Genes within and Near Loci Identified in Genome-wide Association Studies

Genes associated with susceptibility loci were identified based upon the NHGRI genome-wide association study (GWAS) catalogue [82]. As of March 2013, the NHGRI catalogue included 10732 entries listing SNP loci for which variants have been associated with human traits and diseases. Based upon this information, we generated a map associating 7505 genes to 697 human diseases and traits. This map was then filtered to include only skin-expressed genes, yielding a final map associating 4999 skin-expressed genes to 678 human diseases and traits. For a given SNP, an associated human gene was considered to be the candidate reported by authors in the original GWAS publication or any other gene that mapped within or immediately adjacent to the SNP’s genome coordinates. Gene-SNP associations, therefore, included those in which a SNP was located within a gene, as well as cases in which a SNP was located in the upstream or downstream region. Given the final gene-SNP association map, we evaluated whether psoriasis DEGs overlapped significantly with those genes linked to each of 678 human diseases and traits (Fisher’s Exact Test). This procedure was performed separately for the 1068 PP-increased DEGs and the 907 PP-decreased DEGs, and in each case we identified those diseases and traits for which the observed overlap was most significant (Figure S5). To control the false discovery rate (FDR) among tests performed for each of the 680 diseases and traits, raw p-values derived from Fisher’s exact test were adjusted using the Benjamini-Hochberg method [81]. Based on the gene-SNP mapping, we also identified 40 skin-expressed genes associated with psoriasis susceptibility loci. Of these 40 genes, we identified the 30 most significantly altered in PP versus PN skin (*n* = 215 patients; Figure S7).

### Cluster Analysis and Detection of Epidermis Gene Expression Modules

Gene modules were generated by cluster analysis of expression patterns across 149 microarrays in which expression was measured in KCs or epidermal isolates (Affymetrix Human Genome U133 Plus 2.0 Array). The 149 samples were selected from an initial batch of 191 CEL files obtained from Gene Expression Omnibus. Quality control metrics were calculated for each file and we removed from consideration arrays with low or high background (10%), arrays with low or high scale intensity factor (10%), arrays with a low percentage of probe sets called present (5%), and arrays with high RNA degradation score (5%). Based on these criteria, a total of 42 arrays were removed, yielding our final set of 149 KC/epidermis data samples. This final set included samples from both untreated and cytokine-treated primary KCs (109/149), epidermal sheets isolated by ammonium thiocyanate incubation (36/149), and epidermis fractions isolated by laser capture microdissection (4/149). Consistent with differential expression analyses, one representative probe set was analyzed for each human gene (see selection criteria above). Genes were filtered to include only skin- and epidermis-expressed genes, with each gene expressed significantly above background in at least 10% of the 149 KC/epidermis samples and at least 10% of the 430 PP and PN bulk skin biopsy samples. The 5% of genes showing the least variation across the 149 KC/epidermis samples were also removed from consideration. These filtering steps yielded a final set of 13391 skin- and epidermis-expressed genes that were included in clustering analyses.

The 149 KC/epidermis microarray samples were normalized using Robust Multichip Average (RMA). For each gene, expression values associated with the representative probe set were first “centered” to have a mean of zero across all 149 KC/epidermis samples. This yielded an expression profile for each gene with positive values indicating above-average expression and negative values indicating below-average expression (e.g., see Figure 5A). Expression profile values were then standardized, for each array, such that standardized values had a mean of 0 and variance of 1 among all genes. The 13391 genes were then clustered using average linkage hierarchical clustering and the Euclidean distance metric. The function “cutreeDynamicTree” within the R package “dynamictreecut” was used to detect clusters within the resulting dendrogram using a minimally permitted cluster size threshold of 25 genes [83]. This approach detects clusters within a dendrogram at varying heights using an iterative top-down approach, in which clusters are sub-divided until either the minimum cluster size threshold is reached (25 genes) or until height differences of genes within a cluster are no longer significant [83].

### Gene Set Enrichment Analysis (GSEA) and Detection of Differentially Expressed Modules

Gene set enrichment analyses were performed using the rank-based statistical approach described by Philippakis et al. [84]. In this method, genes are ranked according to how their expression is altered in a given experiment (e.g., PP versus PN skin) and the ranks are compared between a foreground and background gene set (e.g., see Figure 3). Statistical enrichment was visually assessed by inspecting the area between “detection rate curves” associated with the foreground and background gene sets [84]. The p-value associated with this area is identical to that obtained by comparison of foreground and background gene ranks using the Wilcoxon rank sum test statistic [84]. GSEA was used to assess whether DEMs were enriched with PP-increased or PP-decreased genes (Figure 2). In this case, the foreground gene set was defined as DEM genes, while the background gene set was defined as all other epidermis- and skin-expressed genes. Genes were ranked based upon the score *I*×–log(p-value), where *I* = 1 if the gene is PP-increased, *I* = -1 if the gene is PP-decreased, and the p-value is generated by the test for differential expression between PP versus PN skin (*n = *215; Wilcoxon rank sum test). In other cases, GSEA was used to assess whether DEM genes were biased towards increased or decreased expression in LCM-dissected PP epidermis (Figure 3), cytokine-treated epidermal cells (Figures 4, 5B and 5C), or cultured KCs following siRNA treatments targeting specific genes (Figures 5D and 5E). In each case, DEM genes were defined as the foreground gene set and all other epidermis- and skin-expressed genes were defined as the background gene set. Genes were then ranked according to the above-described score *I*×–log(p-value), where the p-value was derived from a test for differential expression in LCM-dissected PP epidermis versus LCM-dissected PN epidermis (Figure 3), cytokine-treated epidermal cells versus non-treated control cells (Figures 4, 5B and 5C), or cells treated with targeting siRNA versus scrambled siRNA (Figures 5D and 5E).

### Functional Annotation Analysis of DEM Genes

DEMs were analyzed to determine if member genes were disproportionately associated with functional annotations derived from several sources, including Gene Ontology (GO) [85], Kyoto encyclopedia of genes and genomes (KEGG) [86], Reactome [87] and PharmGKB [88] (Table S2 and Figure S11). Enrichment of GO and KEGG terms among DEM genes was assessed using the conditional hypergeometric test implemented in the “GOstats” R package [89]. Enrichment of Reactome terms among DEM genes was assessed using Fisher’s Exact test and annotation from the “reactome.db” R package. Likewise, enrichment of PharmGKB terms was assessed using Fisher’s Exact test and PharmGKB pathway annotation files [88]. In all analyses, the frequency of annotation terms among DEM genes was compared to the frequency of annotation terms among all other epidermis- and skin-expressed genes.

### Identification of DEMs with Cytokine-responsive Genes

Microarray data from Gene Expression Omnibus was used to identify DEMs with genes induced or repressed by cytokines (Figure 4). These data were generated from primary KCs (GSE440, GSE2489, GSE2822, GSE7216, GSE7661, GSE8531, GSE9120, GSE12109, GSE17892, GSE24767, GSE32620, GSE36287 and GSE36387), HaCaT KCs (GSE27533 and GSE32975), DK7 KCs (GSE1132) or reconstituted epidermis (GSE2822 and GSE25400). In one experiment, data were generated from whole skin biopsies 24 hours following intradermal injection with IFN-γ (GSE32407). For each experiment, cytokine-responsive genes were identified based upon empirical Bayes methods as implemented in the R “limma” package [80]. On most array platforms, multiple probe sets were available to measure expression of a single human gene [79]. For a given human gene, therefore, expression was measured based upon the probe set feature showing the strongest change in gene expression with cytokine treatment (i.e., lowest p-value). For a given experiment, two methods were used to assess whether DEMs were enriched for cytokine-responsive genes. First, genes were ranked based upon their expression shift with cytokine treatment (*I*×–log(p-value)), and GSEA was used to assess whether DEMs were enriched with cytokine-induced or cytokine-repressed genes (see above for description of GSEA). Second, we assessed whether DEM genes overlapped significantly with either the set of cytokine-induced genes or the set of cytokine-repressed genes (P<0.001; Fisher’s Exact Test). A DEM was considered biased towards cytokine-induced or cytokine-repressed expression only if both methods yielded significant p-values (P<0.001), with agreement regarding the direction of effect (i.e., cytokine-induced or cytokine-repressed; Figure 4). Genes considered in these analyses were filtered to include only those among the 13391 epidermis- and skin-expressed genes (see above).

### Identification of DEMs Biased Towards Hypo- or Hyper-methylated Genes

Methylation in lesional (PP) and uninvolved (PN) skin was assessed in 8 patients using data from Roberson et al. [17] (GSE31835) (Figure S12). Methylation in this study was profiled using the Illumina HumanMethylation27 BeadChip array, which features probes for detecting methylation at 27578 genomic sites. For each site, we calculated methylation ratios (*M* values) equal to the log_2_-transformed ratio between methylated and unmethylated signal intensity [17]. Evidence for differential methylation at each site was then assessed based upon 8 paired PP versus PN differences in M values and the moderated empirical Bayes t-test procedure implemented in the R “limma” package [80]. For each human gene, we identified the single site showing the strongest methylation difference between PP and PN skin (i.e., lowest p-value). Human genes were then ranked according to the strength of this methylation difference (*I*×–log(p-value)) and GSEA was used to assess whether DEM genes were biased towards hypo- or hyper-methylation (see above for description of GSEA). Genes considered in these analyses were filtered to include only those among the 13391 epidermis- and skin-expressed genes (see above).

### Additional Microarray Datasets (LCM Samples, Drug Studies, Wound Healing, Non-melanoma Skin Cancers, Glucocorticoid and RNAi Experiments)

Gene expression in LCM-dissected PP epidermis and LCM-dissected PN epidermis was evaluated using data available under GEO accession GSE26866 (Figure 3). We analyzed paired PP and PN samples from 3 patients in which cDNA had been prepared using two-cycle linear amplification and hybridized with Affymetrix Human Genome U133A 2.0 arrays [38]. The effect of etanercept (anti-TNF), ixekizumab (anti-IL-17; previously LY2439821) and efalizumab (anti-CD11a) on gene expression in PP skin was evaluated using data from GSE11903, GSE31652 and GSE30768, respectively (Figure S14). In brief, 50 mg etanercept was given by subcutaneous (sc.) injection twice weekly for 12 weeks (clinical trial no. NCT00116181) [75], two doses of 150 mg ixekizumab were given sc. over 2 weeks [90], and 1 mg/kg efalizumab was given sc. once weekly for 12 weeks (clinical trial no. NCT00115076) [91]. For each drug study, expression was evaluated using the Affymetrix Human Genome U133A 2.0 array platform. Transcriptional effects of wound healing were evaluated using microarray data available under GEO accession GSE8056 [92] (Affymetrix Human Genome U133A 2.0 array). Samples analyzed in our analysis were obtained from burn wound margins of patients at least 7 days following thermal injury [92]. Microarray data from non-melanoma skin cancer biopsies was obtained from GEO series GSE2503 (actinic keratosis and squamous cell carcinoma; Affymetrix Human Genome U133A Array) and GEO series GSE7553 (basal cell carcinoma; Affymetrix Human Genome U133 Plus 2.0 array) [93], [94]. The transcriptional effect of glucocorticoid (dexamethasone) in normal human KCs was evaluated based upon microarray data from GEO series GSE26487 (Affymetrix Human Genome U95 Version 2 Array). In this experiment, KCs at approximately 70% confluence were treated with 0.1 μM dexamethasone for 72 hours prior to harvesting of cellular RNA [95]. The effect of TP63 on gene expression in primary neonatal KCs was evaluated using data available under GEO accession GSE33495 (Figure 5). In these experiments, Affymetrix Human Genome U133 Plus 2.0 arrays were used to compare gene expression in KCs treated with siRNA oligomers directed against *TP63* (*n* = 2) and KCs treated with scrambled control siRNA (*n* = 2). Effects of *ZNF750* and *KLF4* on gene expression in primary neonatal KCs were evaluated using data from GSE32685 (Figure 5). In these experiments, Affymetrix Human Genome U133A 2.0 arrays were used to compare gene expression in KCs treated with siRNA oligomers against ZNF750 or KLF4 (*n* = 2 per group) and KCs treated with a non-targeting control siRNA (*n* = 2) [27]. For each of the above-mentioned datasets, raw expression data was downloaded and normalized using robust multichip average (RMA) [78]. GSEA analyses were performed by ranking skin- and epidermis-expressed genes based upon evidence for differential expression as assessed by the empirical Bayes moderated t-test procedure implemented in the R “limma” package [80].

### Motif Dictionary

We generated a dictionary of 1937 DNA-binding motifs by pooling collections available from five sources, including 135 motifs from the Jaspar (vertebrate) database [96], 283 from the UniPROBE database [97], 290 from the ENCODE Motif Browser [44], 433 from the human protein DNA Interactome (hPDI) database [98], and 796 from the TRANSAC vertebrate collection [99]. To obtain the 1937 motifs, we initially screened a broader collection of 2836 motifs (145 from Jaspar; 296 from UniPROBE; 293 from ENCODE; 437 from hPDI; 1665 from TRANSFAC). A motif was removed if it was less than 4 base pairs in length or if it was redundant with another already in the dictionary. Two motifs were considered redundant if they had the same consensus sequence and if corresponding values in PPM matrices differed by less than 0.02 on average. Approximately half of the motifs from the initial TRANSFAC vertebrate collection were removed because of redundancy with motifs available from one of the other four sources. Following these pre-processing steps, our final dictionary included 1937 non-redundant DNA-binding motifs, which collectively, were associated with proteins derived from 997 unique human genes. Of these 997 genes, approximately 70% (688/997) were annotated with GO BP Terms “Transcription factor activity” (GO:0003700), “DNA binding” (GO:0003677), or “Transcription cofactor activity” (GO:0003712) (Figure S16).

### Semiparametric Generalized Additive Logistic Models and Identification of Motifs Enriched in Genome Regions Near DEM Genes

Associations between motif frequency and DEM genes were evaluated using semiparametric generalized additive logistic models (GAM) as recently described by Swindell et al. [100]. For each of 13391 epidermis- and skin-expressed genes, a response variable was coded 1 if a gene belonged to the DEM and coded 0 if a gene did not belong to the DEM. For each motif, the response variable was used in a GAM logistic regression model with two predictors (*x*_1_ and *x*_2_), where *x*_1_ is equal to the (log-transformed) length of scanned sequence associated with each gene, and *x*_2_ is equal to the (log-transformed) number of motif occurrences within the scanned sequence [100]. The predictor variable representing sequence length (*x*_1_) was incorporated into regression models as a non-parametric smoothed term, while motif frequency (*x*_2_) was incorporated as a parametric term without smoothing. An association between DEM genes and motif frequency was assessed based upon the Z-statistic and p-value associated with the predictor variable representing motif frequency (*x*_2_) [100]. Regression coefficients were estimated by iterative backfitting of weighted additive models, with parametric coefficients estimated by weighted linear least squares, as implemented in the R “gam” package. For each DEM, this modeling approach was used to screen 1937 DNA-binding protein motifs for possible associations between motif frequency and DEM membership. To control the false discovery rate (FDR) among tests performed for each of the 1937 motifs, raw p-values derived from GAM models were adjusted using the Benjamini-Hochberg method [81]. For each gene, motif frequency was evaluated in intergenic regions or upstream sequences (2 KB or 5 KB), and in each case, we masked coding sequences, assembly gaps, and repetitive DNA elements. In some analyses, scanned sequences were further masked to exclude regions weakly conserved among 45 vertebrate genomes. Such regions were identified using base-specific PhastCons scores, which range between 0 and 1 and estimate the probability that a base is located within a conserved element [101]. For a given sequence, we identified the median PhastCons score among all bases and masked bases with PhastCons score less than the median value [100]. However, if the median value was greater than 0.70, then all bases with PhastCons score less than 0.70 were masked [100].

### Immunohistochemistry

Anti-IRF1 and anti-pSTAT1 (Ser727) antibodies were obtained from Cell Signaling Technology (cat#8478 and cat#8826 respectively). Anti-IRF9 antibody was obtained from Thermo Scientific (cat # PA5-30378). Diaminobenzidine staining of paraffin embedded tissue sections of uninvolved and lesional psoriatic skin (*n* = 3 for each) was performed using methods as previously described by our group [100].

## Supporting Information

Figure S1 **Top-ranked genes most strongly increased or decreased in psoriasis lesions (** ***n*** **= 215 patients).** For each patient, expression fold-change (PP/PN) was calculated based upon the comparison between lesional (PP) and uninvolved (PN) skin samples. The chart shows (A) the top 35 PP-increased genes and (B) the top 35 PP-decreased genes (FDR <0.05; ranked by fold-change). For each gene, the grey box spans the middle 50% of fold-change estimates among patients, with whiskers for each box spanning the middle 90% of fold-change estimates. Yellow symbols denote the 5% of patients with extreme fold-change estimates on each side of the fold-change distribution. Median fold-change is listed in the right margin with p-values generated by a non-parametric statistical test (Wilcoxon rank sum test).
(TIF)

Figure S2 **Psoriasis DEGs and their altered expression in six study cohorts (** ***SERPINB4*** **,** ***MMP12*** **,** ***THRSP*** **and** ***PM20D1*** **).** Expression of four psoriasis DEGs was evaluated in the six study cohorts. For each gene and cohort, grey boxes span the middle 50% of fold-change estimates among patients, with whiskers for each box spanning the middle 90% of fold-change estimates. Yellow symbols denote the 5% of patients with extreme fold-change estimates on each side of the fold-change distribution. Median fold-change is listed in the right margin with p-values generated by a non-parametric statistical test (Wilcoxon rank sum test).
(TIF)

Figure S3 **Gene ontology (GO) biological process terms overrepresented among genes with elevated expression in psoriasis lesions.** We identified 1068 DEGs with significantly elevated expression in psoriasis lesions as compared to normal uninvolved skin (FDR <0.05 and FC >1.50). The figure lists top-ranked GO biological process terms disproportionately associated with these DEGs as compared to all other skin-expressed genes (conditional hypergeometric test). Values in parentheses indicate the number of DEGs (out of 1068) associated with each GO term. The right margin lists example DEGs associated with the corresponding GO term.
(TIF)

Figure S4 **Gene ontology (GO) biological process terms overrepresented among genes with decreased expression in psoriasis lesions.** We identified 907 DEGs with significantly decreased expression in psoriasis lesions as compared to normal uninvolved skin (FDR <0.05 and FC <0.67). The figure lists top-ranked GO biological process terms disproportionately associated with these DEGs as compared to all other skin-expressed genes (conditional hypergeometric test). Values in parentheses indicate the number of DEGs (out of 907) associated with each GO term. The right margin lists example DEGs associated with the corresponding GO term.
(TIF)

Figure S5 **Psoriasis DEGs overlap significantly with genes near susceptibility loci for auto-immune diseases (inflammatory bowel disease, Celiac disease and Crohn’s disease).** The figure lists traits for which genes near susceptibility loci overlap most significantly with (A) 1068 PP-increased DEGs and (B) 907 PP-decreased DEGs. Top-ranked traits are listed in the left margin. Genes associated with susceptibility loci were identified based upon the NHGRI genome-wide association study catalogue. For each trait, we tested for significant overlap between trait-associated genes and psoriasis DEGs using Fisher’s Exact Test. Values in parentheses indicate the total number of DEGs associated with the listed trait. The right margin lists example DEGs associated with the listed trait and included among the (A) 1068 PP-increased DEGs and (B) 907 PP-decreased DEGs. The dark grey background region in (A) and (B) denotes P<0.05 (Fisher’s Exact Test; magenta bars are significant with FDR <0.05).
(TIF)

Figure S6 **Overlap between PP-increased DEGs and genes associated with susceptibility loci for Inflammatory bowel, Celiac and Crohn’s disease.** PP-increased DEGs overlapped significantly with susceptibility loci for inflammatory bowel disease, celiac disease and Crohn’s disease (P≤4.8×10^−5^; FDR ≤0.007; Fisher’s Exact Test). The Venn diagram shows gene counts associated with the intersection of PP-increased genes and the genes near susceptibility loci for each of these three conditions.
(TIF)

Figure S7 **Genes associated with psoriasis susceptibility loci and their expression in psoriasis lesions (** ***n*** **= 215 patients).** Genes associated with psoriasis susceptibility loci were identified from the NHGRI genome-wide association studies catalogue. The chart lists 30 genes associated with susceptibility loci that showed the most significant expression difference in PP versus PN skin. These 30 genes were selected from a total of 40 skin-expressed genes associated with susceptibility loci. The left margin lists the candidate gene, its relation to the susceptibility locus (e.g., intergenic, intron, etc.), and the associated chromosomal region. The right margin lists the estimated median fold-change (PP/PN) and p-value (Wilcoxon rank sum test). For each gene, the grey box spans the middle 50% of fold-change estimates among 215 patients, while box whiskers span the middle 90% of estimates. Yellow symbols denote the 5% of patients with extreme fold-change estimates on each side of the fold-change distribution.
(TIF)

Figure S8 **Epidermal transcription modules most enriched with psoriasis DEGs.** We identified 235 gene modules based upon co-expression patterns in KC and epidermis microarray samples. From among these 235 modules, we identified (A) the 30 modules most enriched with PP-increased DEGs and (B) the 30 modules most enriched with PP-decreased DEGs. Module labels are listed in the left margin with p-value from a test for overrepresentation of PP-increased or PP-decreased genes (Fisher’s Exact Test; P<0.05, one asterisk; FDR <0.05, two asterisks). In (A), symbols indicate the proportion of PP-increased DEGs in each module (± one standard error). In (B), symbols indicate the proportion of PP-decreased DEGs in each module (± one standard error). The yellow background region includes those proportion values greater than the overall proportion of (A) PP-increased or (B) PP-decreased DEGs among skin- and epidermis-expressed genes. The right margin lists psoriasis DEGs belonging to the indicated module that are most strongly (A) increased in PP skin or (B) decreased in PP skin.
(TIF)

Figure S9 **The 30 DEMs show correlated expression in KCs and epidermis.** (A) DEM similarity matrix based upon the Spearman correlation coefficient estimates between module medoids (KC and epidermis microarray samples). Red labels denote DEMs biased towards PP-increased expression, while blue labels denote DEMs biased towards PP-decreased expression (FDR <0.05 by GSEA with median FC >1.25 or median FC <0.80). (B) DEM correlation network (Spearman correlation coefficient). Red lines connect positively correlated DEM pairs and blue lines connect negatively correlated DEM pairs. The thickness of each line is proportional to the absolute magnitude of the Spearman correlation coefficient estimate.
(TIF)

Figure S10 **PP-increased and PP-decreased DEMs localize to distinct regions in principal component space.** The 235 modules were generated by clustering genes based upon expression patterns across 149 KC and microarray samples. To visualize module relationships, the 149 conditions were reduced to two dimensions using principal components (PC) analysis. Modules were then plotted with respect to the two PCs based upon the median PC scores among genes belonging to each module. PP-increased DEMs are shown with red symbols, while PP-decreased DEMs are shown with blue symbols. Dotted red lines denote mean PC values among PP-increased DEMs, while dotted blue lines denote mean PC values among PP-decreased DEMs. Mean PC scores for PP-increased and PP-decreased DEMs differed significantly with respect to both PC axes (P≤4.1×10^−3^; two-sample t-test).
(TIF)

Figure S11 **Psoriasis DEMs are functionally associated with immunity, mitosis, lipids, RNA processing, development/differentiation and apoptosis.** The 30 DEMs were each analyzed to identify significantly overrepresented annotation terms (e.g., Gene Ontology, KEGG, Reactome and PharmGKB databases). Analysis of the observed trends revealed that DEMs were broadly associated with immunity, mitosis, lipids, RNA processing, development/differentiation and apoptosis. The table lists DEMs associated with each category, with off-diagonal entries representing DEMs associated with more than one category. Modules in red font are biased towards PP-increased expression and modules in blue font are biased towards PP-decreased expression.
(TIFF)

Figure S12 **Identification of 3 DEMs biased towards hyper- or hypo-methylation in psoriasis lesions.** The 30 DEMs were evaluated to determine if member genes were disproportionately hyper- or hypo-methylated in PP skin. (A) GSEA detection rate curve area statistics for each of the 30 DEMs (red labels, PP-increased DEMs; blue labels, PP-decreased DEMs). Significant statistics are denoted by an asterisk symbol (P<0.05, black asterisk). Parts (B) and (C) show GSEA results for PTEN-28 and WNT5A-48, respectively. For each human gene, the genomic site showing the strongest methylation difference between PP and PN skin was identified (i.e., lowest p-value). Human genes were then ranked according to the PP versus PN methylation difference estimated at this site (horizontal axis). Low ranks were assigned to genes hyper-methylated in PP skin (left, red region), while high ranks were assigned to genes hypo-methylated in PP skin (right, blue region). Yellow hash marks (top) denote placement of DEM genes with respect to each ranking, and the curve in each figure tracks the cumulative overlap of DEM genes from left to right (vertical axis). Enrichment of DEM genes among genes *hyper*-methylated in PP skin is indicated by a cumulative overlap curve above the diagonal (i.e., positive area statistic; Figure B). Enrichment of DEM genes among genes *hypo*-methylated in PP skin is indicated by a cumulative overlap curve below the diagonal (i.e., negative area statistic; Figure C).
(TIF)

Figure S13 **Expression of STAT1-57 genes in lesional (PP) versus uninvolved (PN) skin from psoriasis patients (** ***n*** **= 215).** The figure shows 40 genes from STAT1-57 with the most significant expression difference in PP versus PN skin (i.e., lowest p-value). Genes were clustered based upon their expression pattern in KC and epidermis microarray samples (Euclidean distance). For each gene, fold-change estimates (PP/PN) among the 215 patients were sorted and displayed in the figure (see color scale). The right margin lists the median fold-change with p-value from a non-parametric test for differential expression between PP and PN skin (Wilcoxon rank sum test).
(TIF)

Figure S14 **STAT1-57 is a glucocorticoid-inhibited DEM repressed in lesions following biologic therapy (etanercept, ixekizumab and efalizumab).** Figures (A) – (D) show GSEA results evaluating whether the STAT1-57 genes are disproportionately increased or decreased in (A) PP lesions following 12 weeks of etanercept treatment versus PP lesions at baseline (GSE11903), (B) PP lesions following 2 weeks of ixekizumab treatment versus PP lesions at baseline (GSE31652), (C) PP lesions following 12 weeks of efalizumab treatment versus PP lesions at baseline (GSE30768) or (D) KCs treated with glucocorticoid (dexamethasone) versus untreated KCs (GSE26487). In each figure (A – D), genes were first ranked according to how their expression is altered in the indicated comparison (horizontal label). Red background denotes genes increased in each comparison while blue background denotes genes decreased in each comparison. Yellow hash marks (top) denote placement of the STAT1-57 genes with respect to each ranking, and the curve in each figure tracks the cumulative overlap of STAT1-57 genes with top-ranked genes from left to right (vertical axis). Enrichment of STAT1-57 members among genes decreased in each comparison is indicated by a cumulative overlap curve below the diagonal (i.e., negative area; Figures A – D).
(TIF)

Figure S15 **PRISMA flowchart for meta-analysis of psoriasis gene expression data.** We followed Preferred Reporting Items for Systematic Reviews and Meta-Analyses (PRISMA) guidelines to evaluate gene expression in lesional (PP) and uninvolved (PN) skin from 215 psoriasis patients. The PRISMA flowchart provides an overview of the identification and screening steps followed to generate data from the 215 patients. In brief, we analyzed all studies that utilized the Affymetrix Human Genome U133 Plus 2.0 microarray platform (GEO accessions: GSE13355, GSE14905, GSE30999, GSE34248, GSE41662 and GSE41663), but excluded four studies in which less comprehensive microarray platforms had been utilized (GEO accessions: GSE2737, GSE6710, GSE11903 and GSE26866).
(PDF)

Figure S16 **Gene ontology (GO) biological process (BP) terms and the 997 human genes associated with DNA motifs included within our dictionary.** The 1937 DNA-binding motifs included within our dictionary were associated with 997 unique human genes. The Venn diagram shows the intersection of these genes with the complete set of human genes annotated with the GO BP terms “Transcription factor activity” (GO:0003700), “DNA binding” (GO:0003677), and “Transcription cofactor activity” (GO:0003712).
(TIF)

Table S1 **Description of cohorts used for microarray datasets (** ***n*** **= 215 patients).** The first column lists the Gene Expression Omnibus accession identifier under which raw data can be accessed. Label refers to the type of target nucleic acid (cRNA or cDNA) used for hybridization with the oligonucleotide array platform (Affymetrix Human Genome U133 Plus 2.0 array). Cohort characteristics are summarized in the final column based upon information provided in original research reports (see footnotes).
(PDF)

Table S2 **Differentially expressed modules (DEMs) biased towards increased or decreased expression in psoriasis lesions.** The table lists genes belonging to each DEM and annotations overrepresented with respect to each DEM (i.e., Gene Ontology, KEGG, Reactome and PharmGKB). Motifs occurring with increased frequency in sequences near DEM genes are also listed (1 KB upstream, 2 KB upstream, 5 KB upstream and all non-coding intergenic regions).
(XLSX)

Checklist S1 **PRISMA checklist for meta-analysis of psoriasis gene expression data.** We followed Preferred Reporting Items for Systematic Reviews and Meta-Analyses (PRISMA) guidelines to evaluate gene expression in lesional (PP) and uninvolved (PN) skin from 215 psoriasis patients. The PRISMA checklist provides an overview of steps followed in our systematic analysis of published gene expression data. In brief, we analyzed all studies that utilized the Affymetrix Human Genome U133 Plus 2.0 microarray platform (GEO accessions: GSE13355, GSE14905, GSE30999, GSE34248, GSE41662 and GSE41663), but excluded four studies in which less comprehensive microarray platforms had been utilized (GEO accessions: GSE2737, GSE6710, GSE11903 and GSE26866).
(PDF)
