# Supplementary figures and images for: Modulation of Epidermal Transcription Circuits in Psoriasis: New Links between Inflammation and Hyperproliferation
Source: PLoS One. 2013 Nov 15;8(11):e79253. doi: 10.1371/journal.pone.0079253 (PMC3829857; doi:10.1371/journal.pone.0079253)

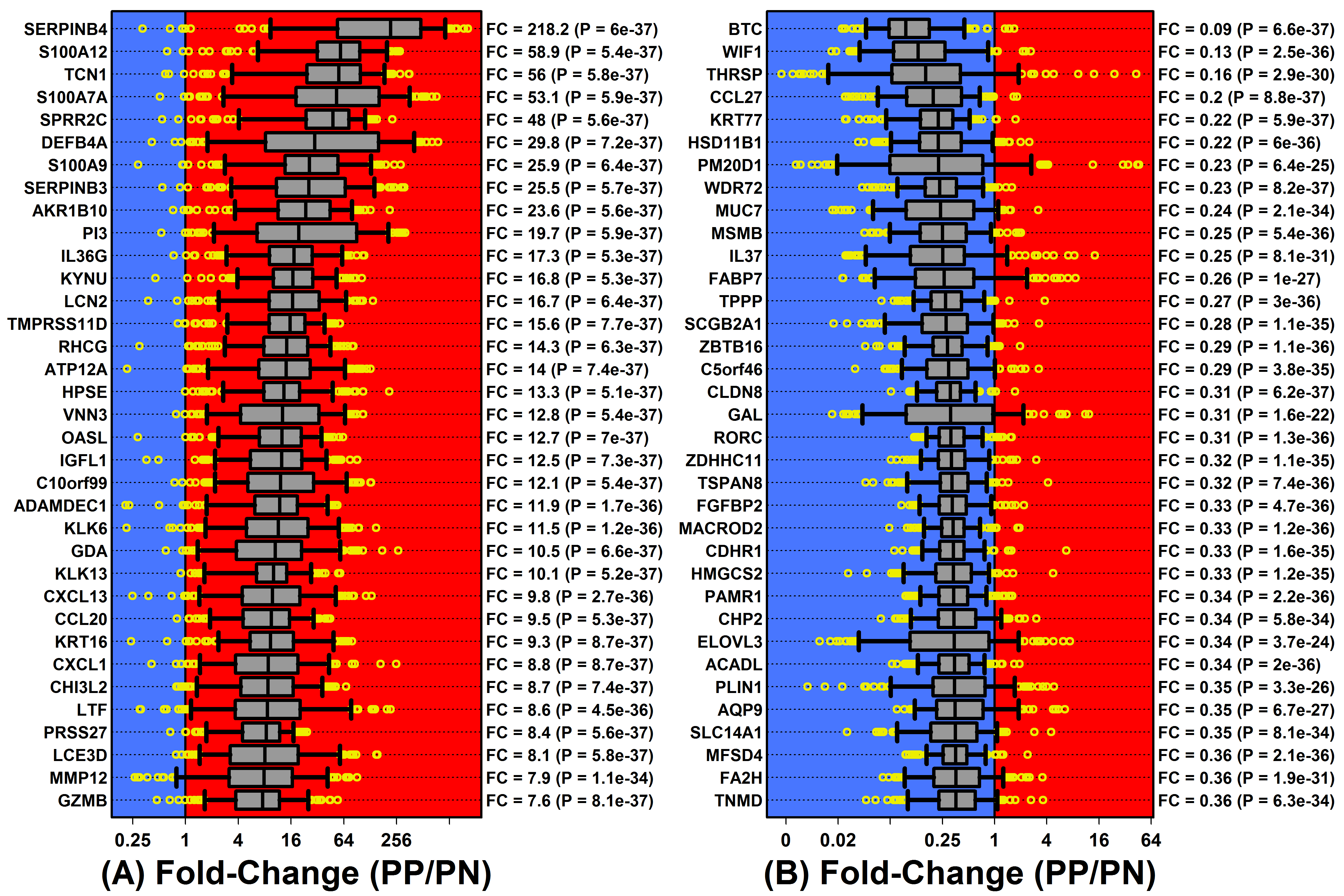

Supplement: Figure S1 — Top-ranked genes most strongly increased or decreased in psoriasis lesions ( n = 215 patients). For each patient, expression fold-change (PP/PN) was calculated based upon the comparison between lesional (PP) and uninvolved (PN) skin samples. The chart shows (A) the top 35 PP-increased genes and (B) the top 35 PP-decreased genes (FDR <0.05; ranked by fold-change). For each gene, the grey box spans the middle 50% of fold-change estimates among patients, with whiskers for each box spanning the middle 90% of fold-change estimates. Yellow symbols denote the 5% of patients with extreme fold-change estimates on each side of the fold-change distribution. Median fold-change is listed in the right margin with p-values generated by a non-parametric statistical test (Wilcoxon rank sum test). (TIF) [file pone.0079253.s001.tif]

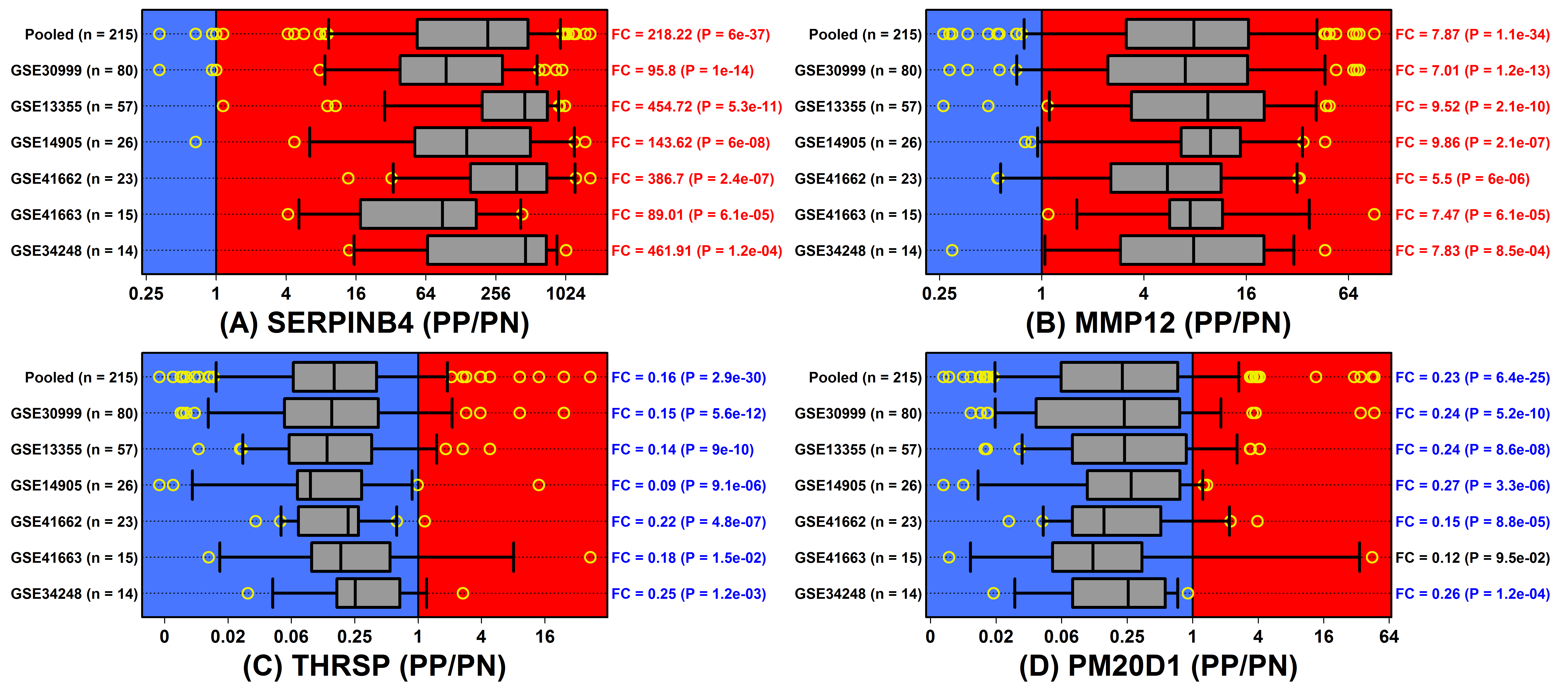

Supplement: Figure S2 — Psoriasis DEGs and their altered expression in six study cohorts ( SERPINB4 , MMP12 , THRSP and PM20D1 ). Expression of four psoriasis DEGs was evaluated in the six study cohorts. For each gene and cohort, grey boxes span the middle 50% of fold-change estimates among patients, with whiskers for each box spanning the middle 90% of fold-change estimates. Yellow symbols denote the 5% of patients with extreme fold-change estimates on each side of the fold-change distribution. Median fold-change is listed in the right margin with p-values generated by a non-parametric statistical test (Wilcoxon rank sum test). (TIF) [file pone.0079253.s002.tif]

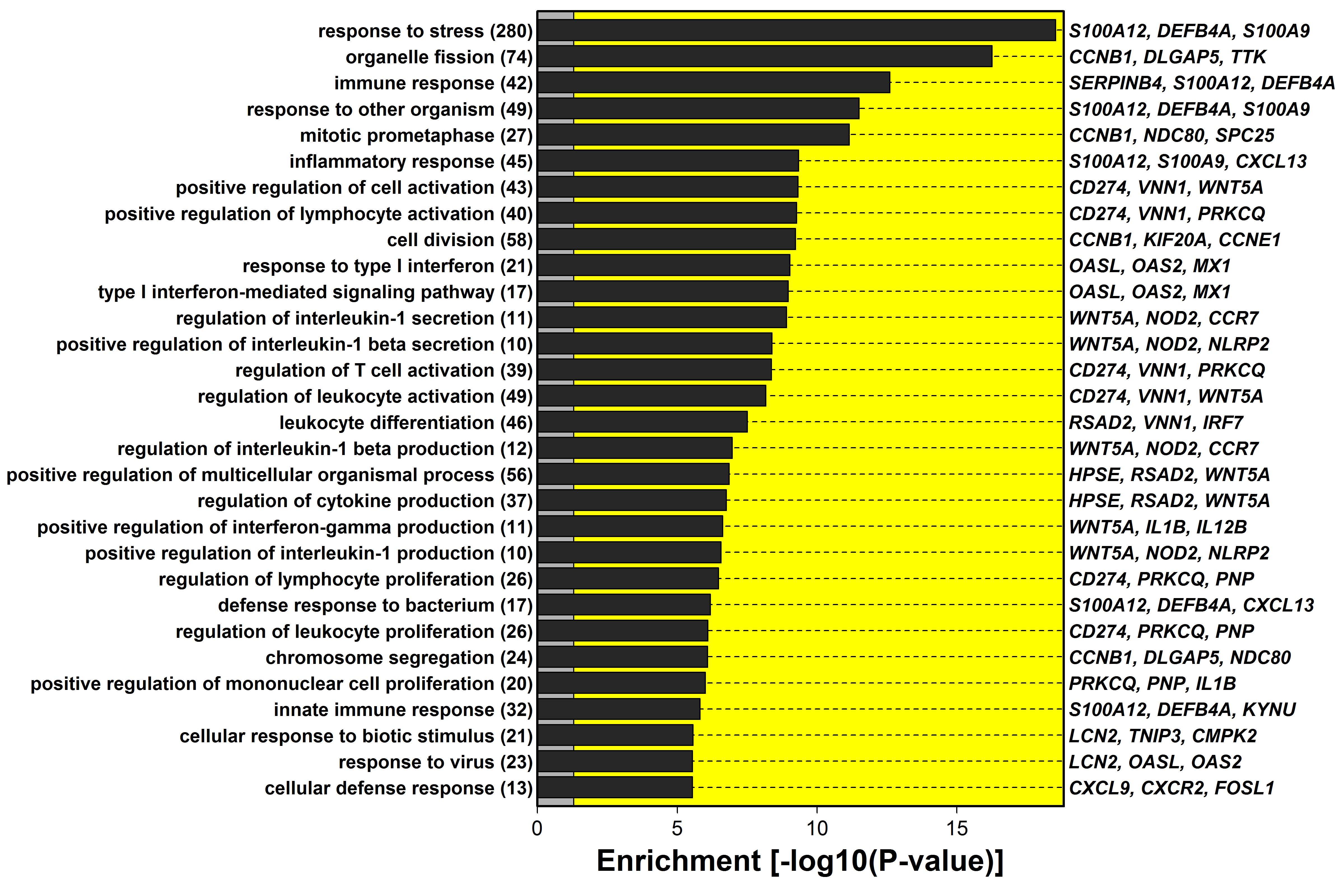

Supplement: Figure S3 — Gene ontology (GO) biological process terms overrepresented among genes with elevated expression in psoriasis lesions. We identified 1068 DEGs with significantly elevated expression in psoriasis lesions as compared to normal uninvolved skin (FDR <0.05 and FC >1.50). The figure lists top-ranked GO biological process terms disproportionately associated with these DEGs as compared to all other skin-expressed genes (conditional hypergeometric test). Values in parentheses indicate the number of DEGs (out of 1068) associated with each GO term. The right margin lists example DEGs associated with the corresponding GO term. (TIF) [file pone.0079253.s003.tif]

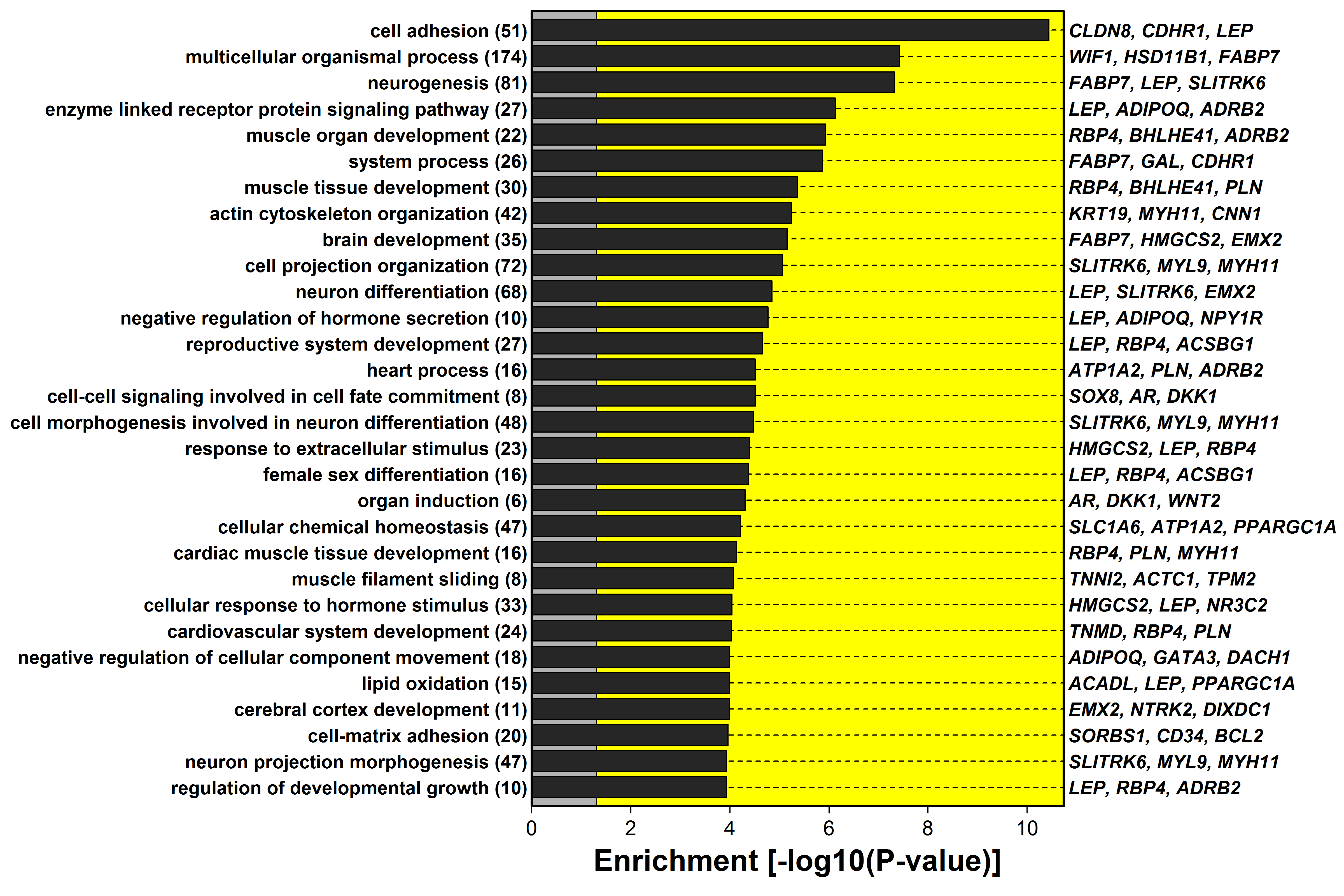

Supplement: Figure S4 — Gene ontology (GO) biological process terms overrepresented among genes with decreased expression in psoriasis lesions. We identified 907 DEGs with significantly decreased expression in psoriasis lesions as compared to normal uninvolved skin (FDR <0.05 and FC <0.67). The figure lists top-ranked GO biological process terms disproportionately associated with these DEGs as compared to all other skin-expressed genes (conditional hypergeometric test). Values in parentheses indicate the number of DEGs (out of 907) associated with each GO term. The right margin lists example DEGs associated with the corresponding GO term. (TIF) [file pone.0079253.s004.tif]

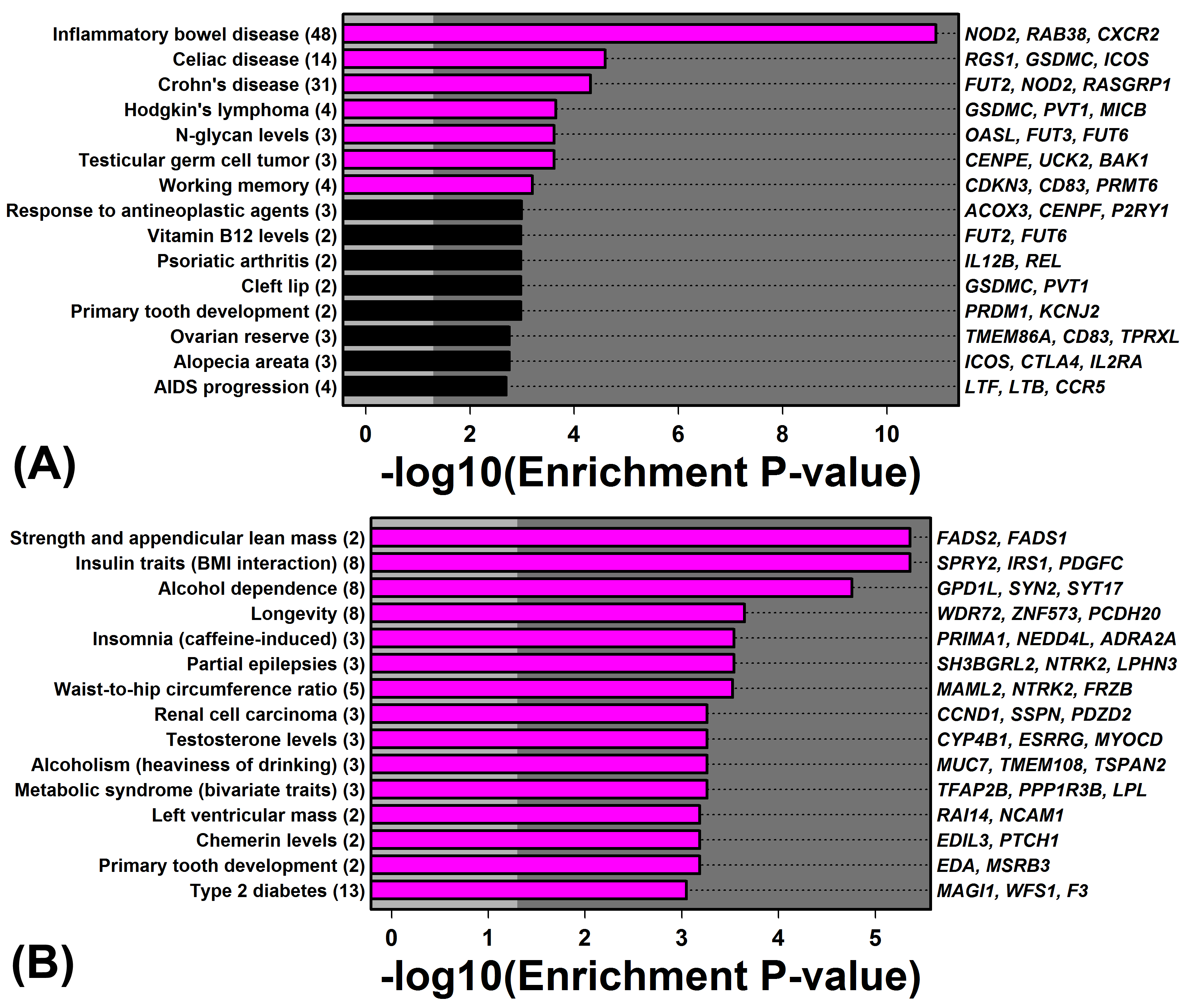

Supplement: Figure S5 — Psoriasis DEGs overlap significantly with genes near susceptibility loci for auto-immune diseases (inflammatory bowel disease, Celiac disease and Crohn’s disease). The figure lists traits for which genes near susceptibility loci overlap most significantly with (A) 1068 PP-increased DEGs and (B) 907 PP-decreased DEGs. Top-ranked traits are listed in the left margin. Genes associated with susceptibility loci were identified based upon the NHGRI genome-wide association study catalogue. For each trait, we tested for significant overlap between trait-associated genes and psoriasis DEGs using Fisher’s Exact Test. Values in parentheses indicate the total number of DEGs associated with the listed trait. The right margin lists example DEGs associated with the listed trait and included among the (A) 1068 PP-increased DEGs and (B) 907 PP-decreased DEGs. The dark grey background region in (A) and (B) denotes P<0.05 (Fisher’s Exact Test; magenta bars are significant with FDR <0.05). (TIF) [file pone.0079253.s005.tif]

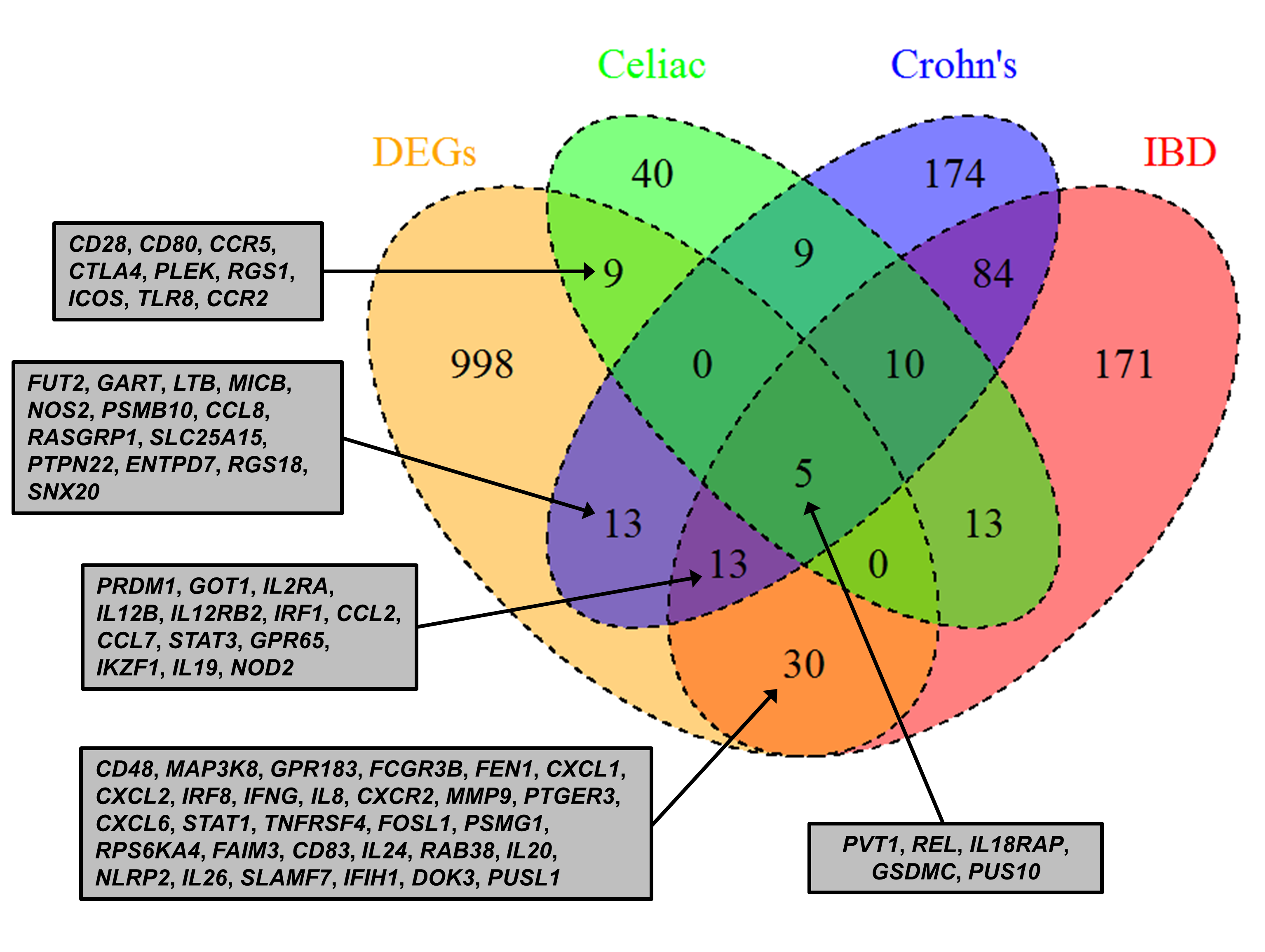

Supplement: Figure S6 — Overlap between PP-increased DEGs and genes associated with susceptibility loci for Inflammatory bowel, Celiac and Crohn’s disease. PP-increased DEGs overlapped significantly with susceptibility loci for inflammatory bowel disease, celiac disease and Crohn’s disease (P≤4.8×10^−5^; FDR ≤0.007; Fisher’s Exact Test). The Venn diagram shows gene counts associated with the intersection of PP-increased genes and the genes near susceptibility loci for each of these three conditions. (TIF) [file pone.0079253.s006.tif]

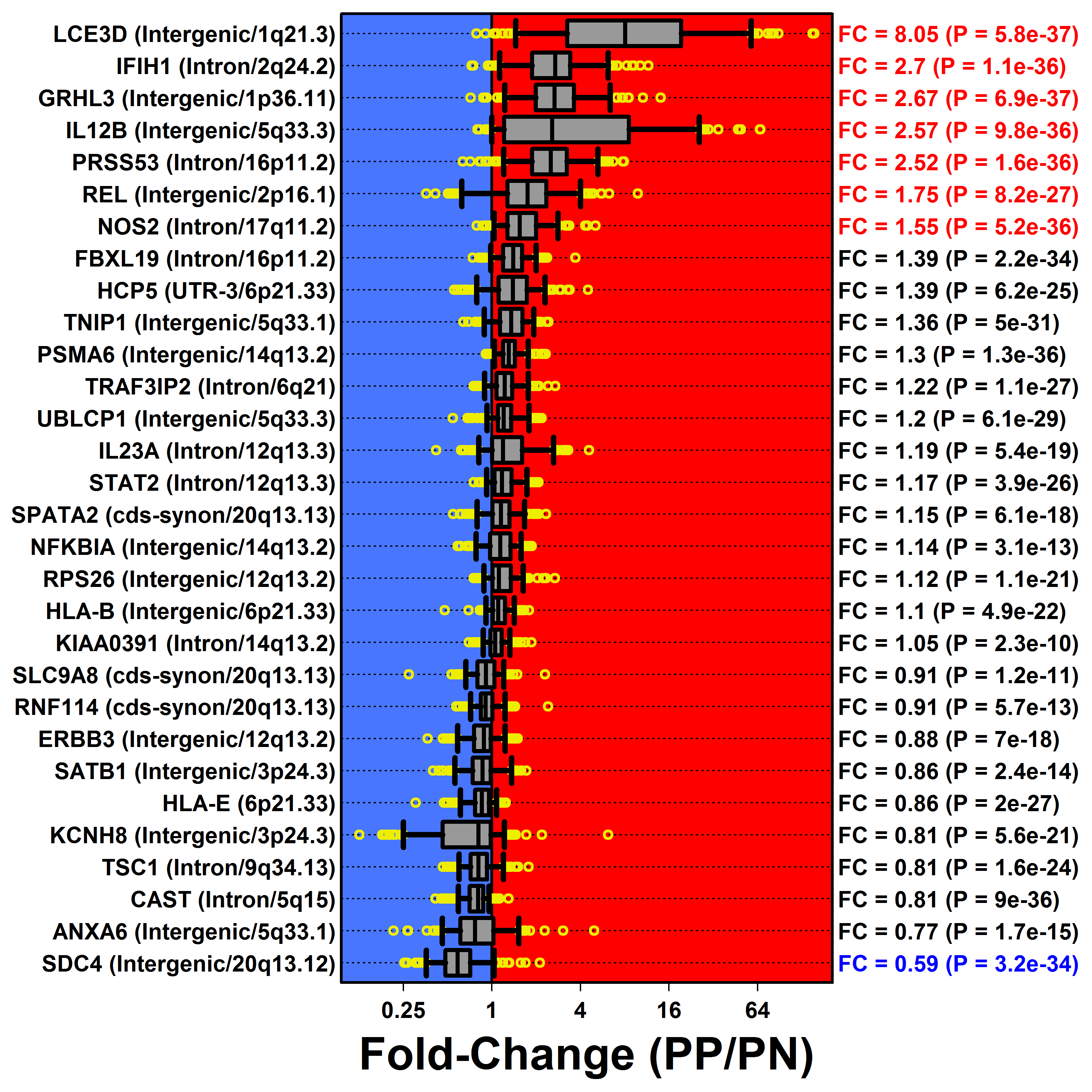

Supplement: Figure S7 — Genes associated with psoriasis susceptibility loci and their expression in psoriasis lesions ( n = 215 patients). Genes associated with psoriasis susceptibility loci were identified from the NHGRI genome-wide association studies catalogue. The chart lists 30 genes associated with susceptibility loci that showed the most significant expression difference in PP versus PN skin. These 30 genes were selected from a total of 40 skin-expressed genes associated with susceptibility loci. The left margin lists the candidate gene, its relation to the susceptibility locus (e.g., intergenic, intron, etc.), and the associated chromosomal region. The right margin lists the estimated median fold-change (PP/PN) and p-value (Wilcoxon rank sum test). For each gene, the grey box spans the middle 50% of fold-change estimates among 215 patients, while box whiskers span the middle 90% of estimates. Yellow symbols denote the 5% of patients with extreme fold-change estimates on each side of the fold-change distribution. (TIF) [file pone.0079253.s007.tif]

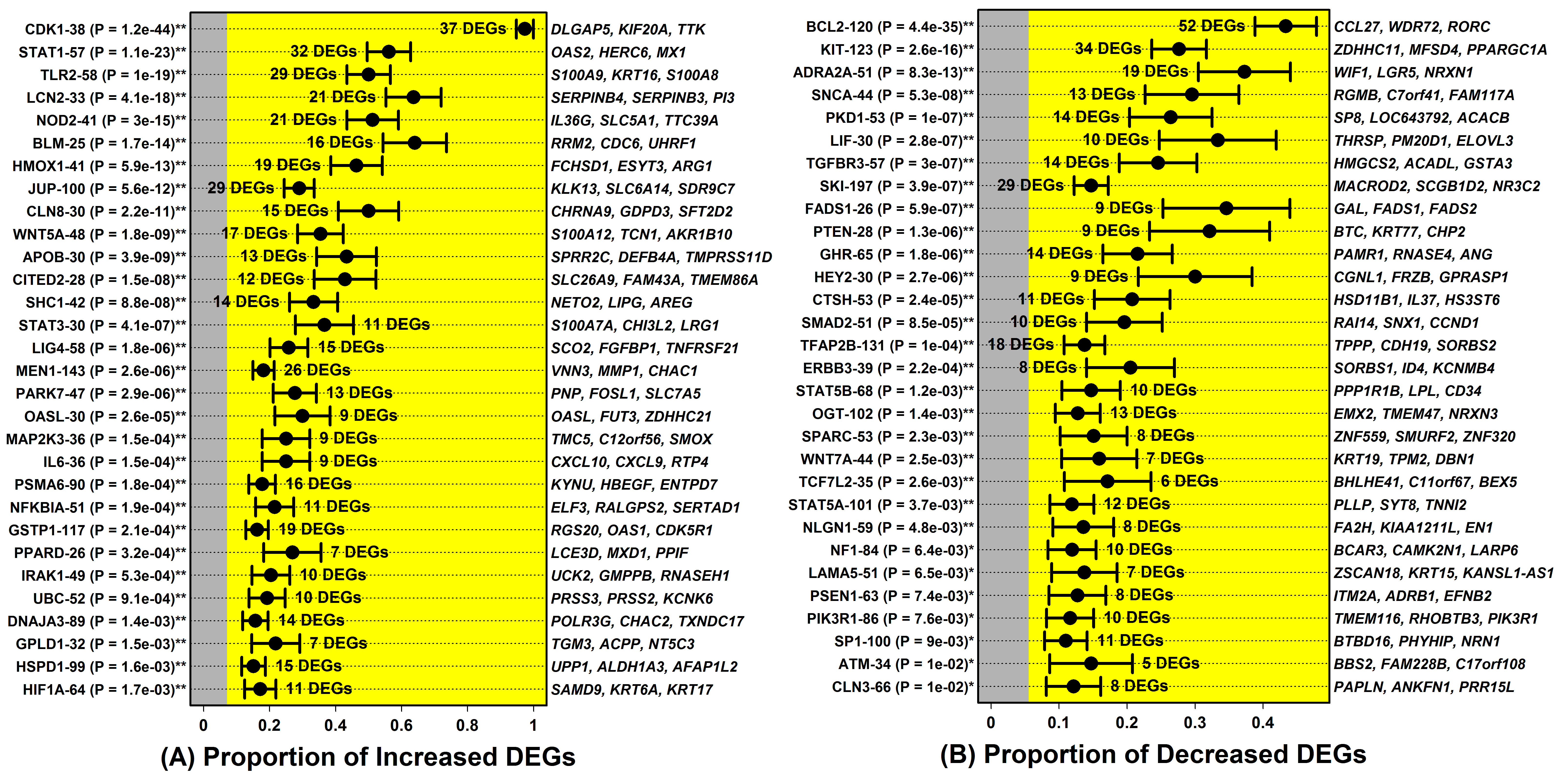

Supplement: Figure S8 — Epidermal transcription modules most enriched with psoriasis DEGs. We identified 235 gene modules based upon co-expression patterns in KC and epidermis microarray samples. From among these 235 modules, we identified (A) the 30 modules most enriched with PP-increased DEGs and (B) the 30 modules most enriched with PP-decreased DEGs. Module labels are listed in the left margin with p-value from a test for overrepresentation of PP-increased or PP-decreased genes (Fisher’s Exact Test; P<0.05, one asterisk; FDR <0.05, two asterisks). In (A), symbols indicate the proportion of PP-increased DEGs in each module (± one standard error). In (B), symbols indicate the proportion of PP-decreased DEGs in each module (± one standard error). The yellow background region includes those proportion values greater than the overall proportion of (A) PP-increased or (B) PP-decreased DEGs among skin- and epidermis-expressed genes. The right margin lists psoriasis DEGs belonging to the indicated module that are most strongly (A) increased in PP skin or (B) decreased in PP skin. (TIF) [file pone.0079253.s008.tif]

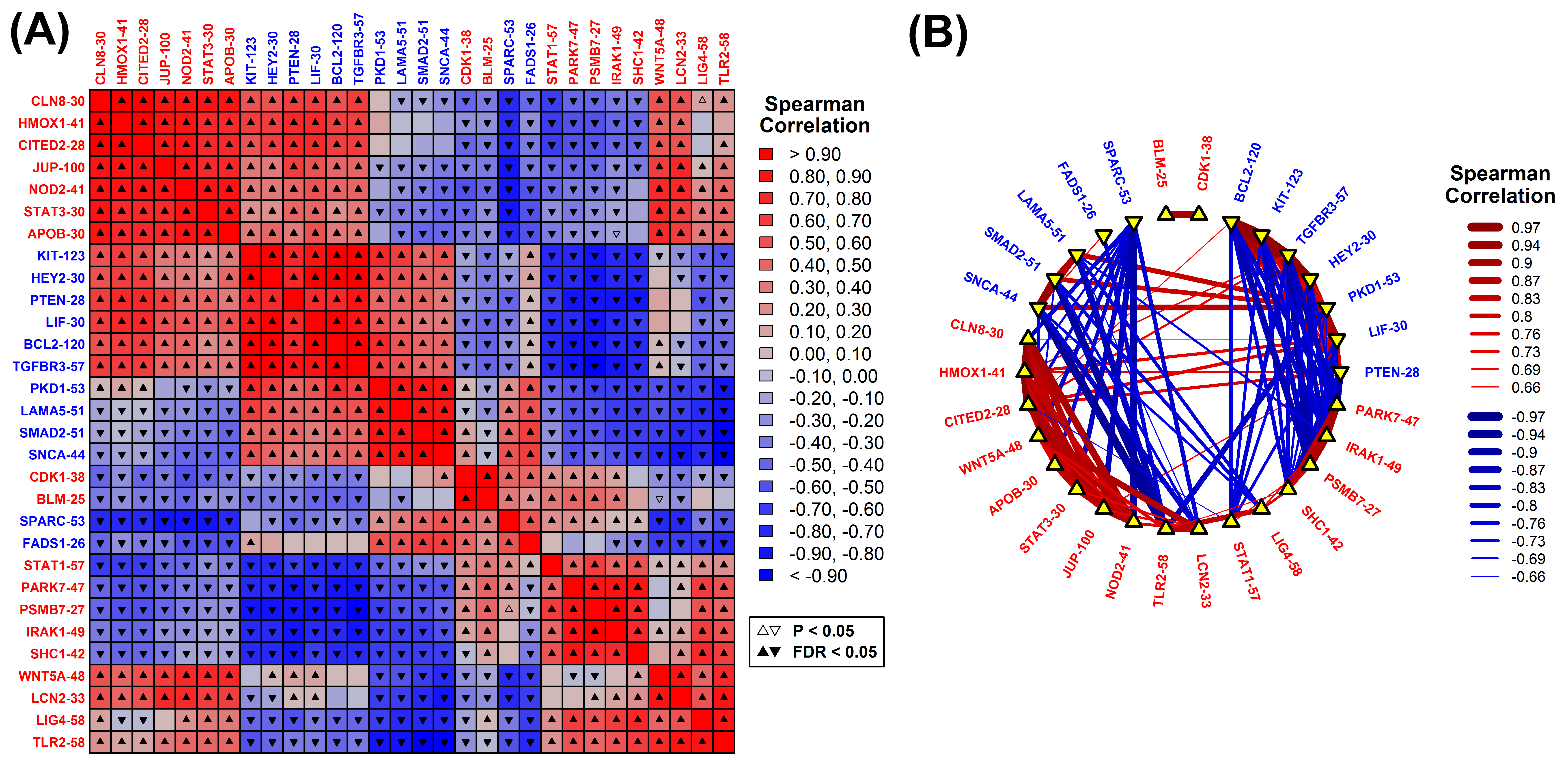

Supplement: Figure S9 — The 30 DEMs show correlated expression in KCs and epidermis. (A) DEM similarity matrix based upon the Spearman correlation coefficient estimates between module medoids (KC and epidermis microarray samples). Red labels denote DEMs biased towards PP-increased expression, while blue labels denote DEMs biased towards PP-decreased expression (FDR <0.05 by GSEA with median FC >1.25 or median FC <0.80). (B) DEM correlation network (Spearman correlation coefficient). Red lines connect positively correlated DEM pairs and blue lines connect negatively correlated DEM pairs. The thickness of each line is proportional to the absolute magnitude of the Spearman correlation coefficient estimate. (TIF) [file pone.0079253.s009.tif]

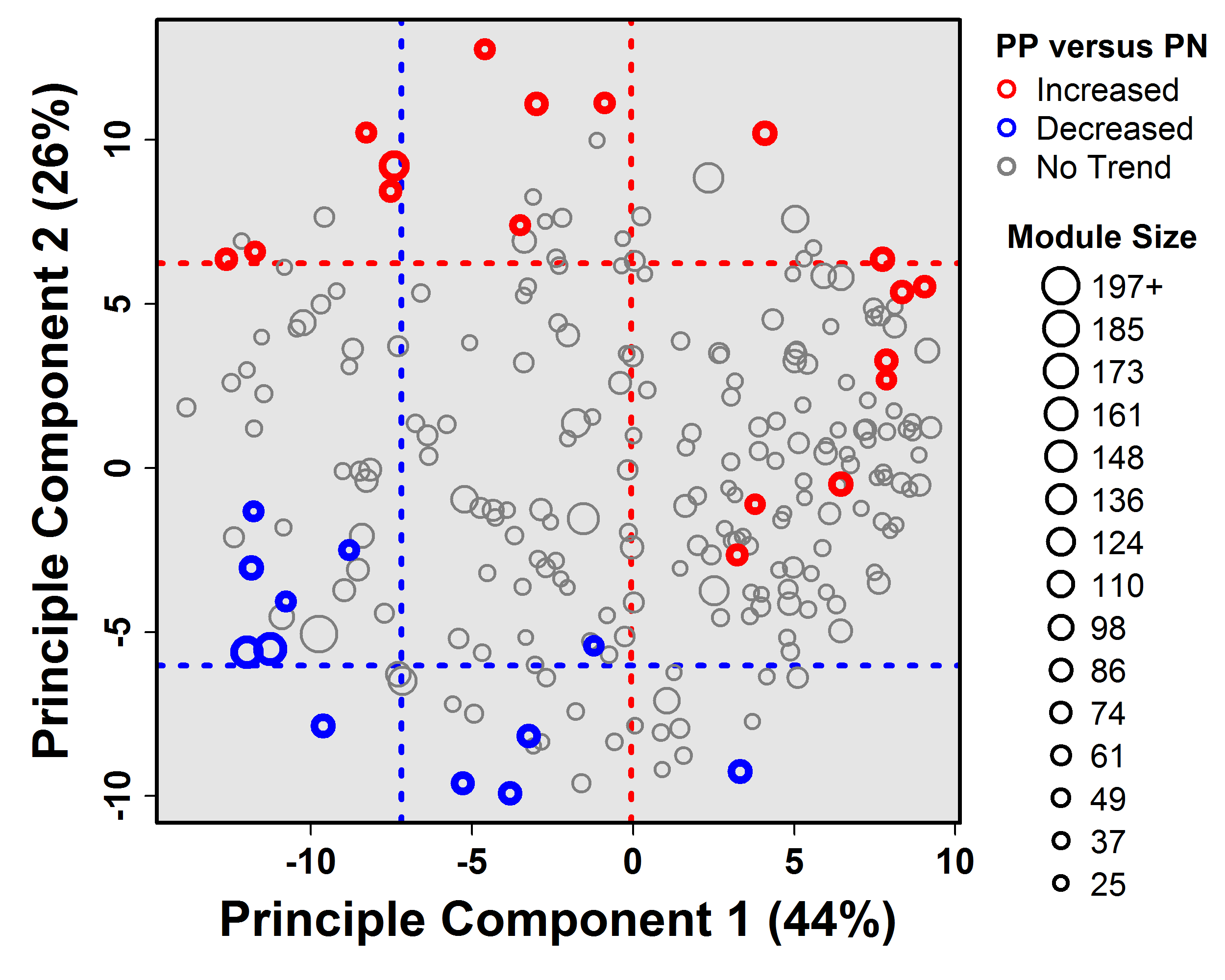

Supplement: Figure S10 — PP-increased and PP-decreased DEMs localize to distinct regions in principal component space. The 235 modules were generated by clustering genes based upon expression patterns across 149 KC and microarray samples. To visualize module relationships, the 149 conditions were reduced to two dimensions using principal components (PC) analysis. Modules were then plotted with respect to the two PCs based upon the median PC scores among genes belonging to each module. PP-increased DEMs are shown with red symbols, while PP-decreased DEMs are shown with blue symbols. Dotted red lines denote mean PC values among PP-increased DEMs, while dotted blue lines denote mean PC values among PP-decreased DEMs. Mean PC scores for PP-increased and PP-decreased DEMs differed significantly with respect to both PC axes (P≤4.1×10^−3^; two-sample t-test). (TIF) [file pone.0079253.s010.tif]

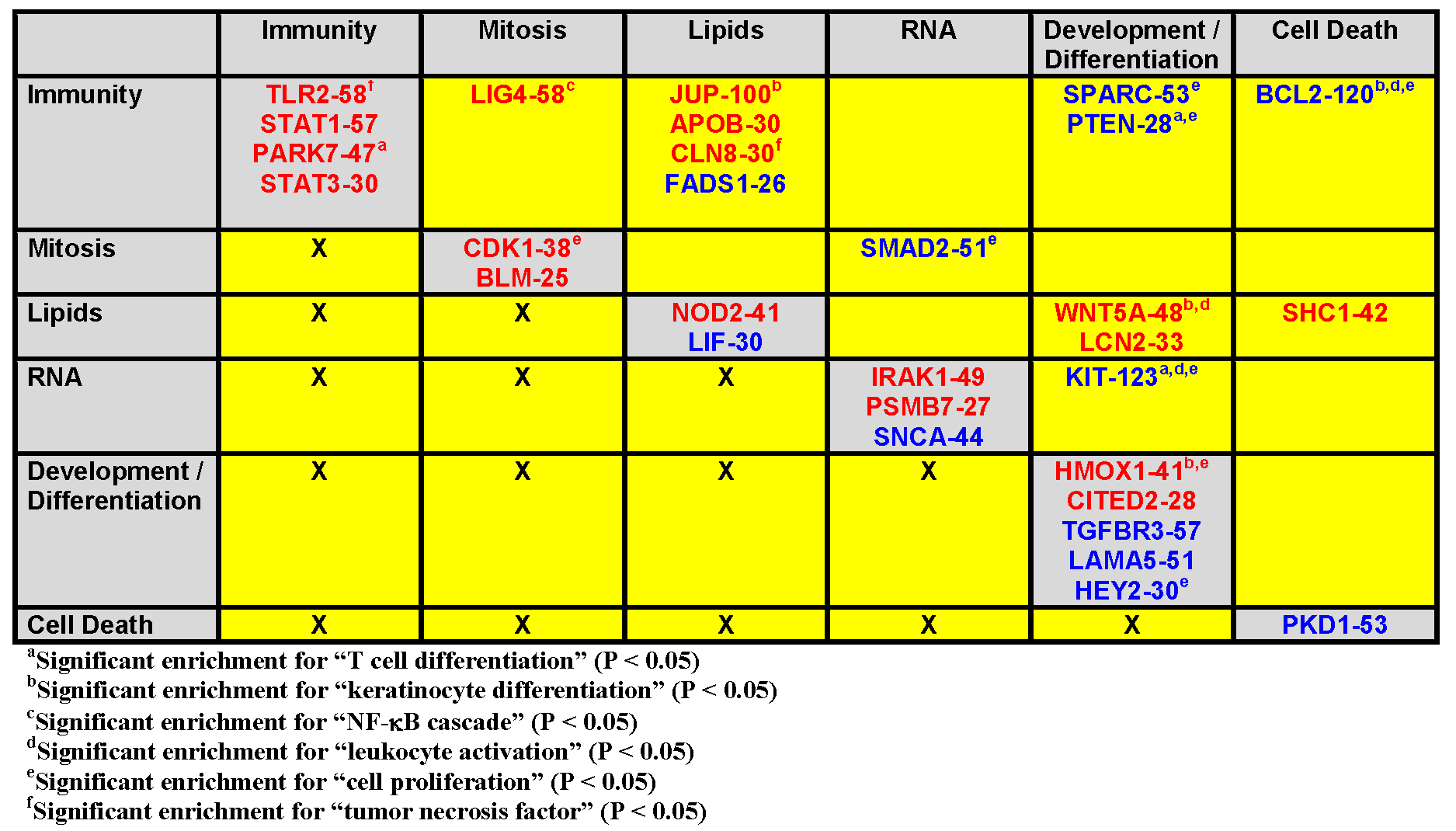

Supplement: Figure S11 — Psoriasis DEMs are functionally associated with immunity, mitosis, lipids, RNA processing, development/differentiation and apoptosis. The 30 DEMs were each analyzed to identify significantly overrepresented annotation terms (e.g., Gene Ontology, KEGG, Reactome and PharmGKB databases). Analysis of the observed trends revealed that DEMs were broadly associated with immunity, mitosis, lipids, RNA processing, development/differentiation and apoptosis. The table lists DEMs associated with each category, with off-diagonal entries representing DEMs associated with more than one category. Modules in red font are biased towards PP-increased expression and modules in blue font are biased towards PP-decreased expression. (TIFF) [file pone.0079253.s011.tif]

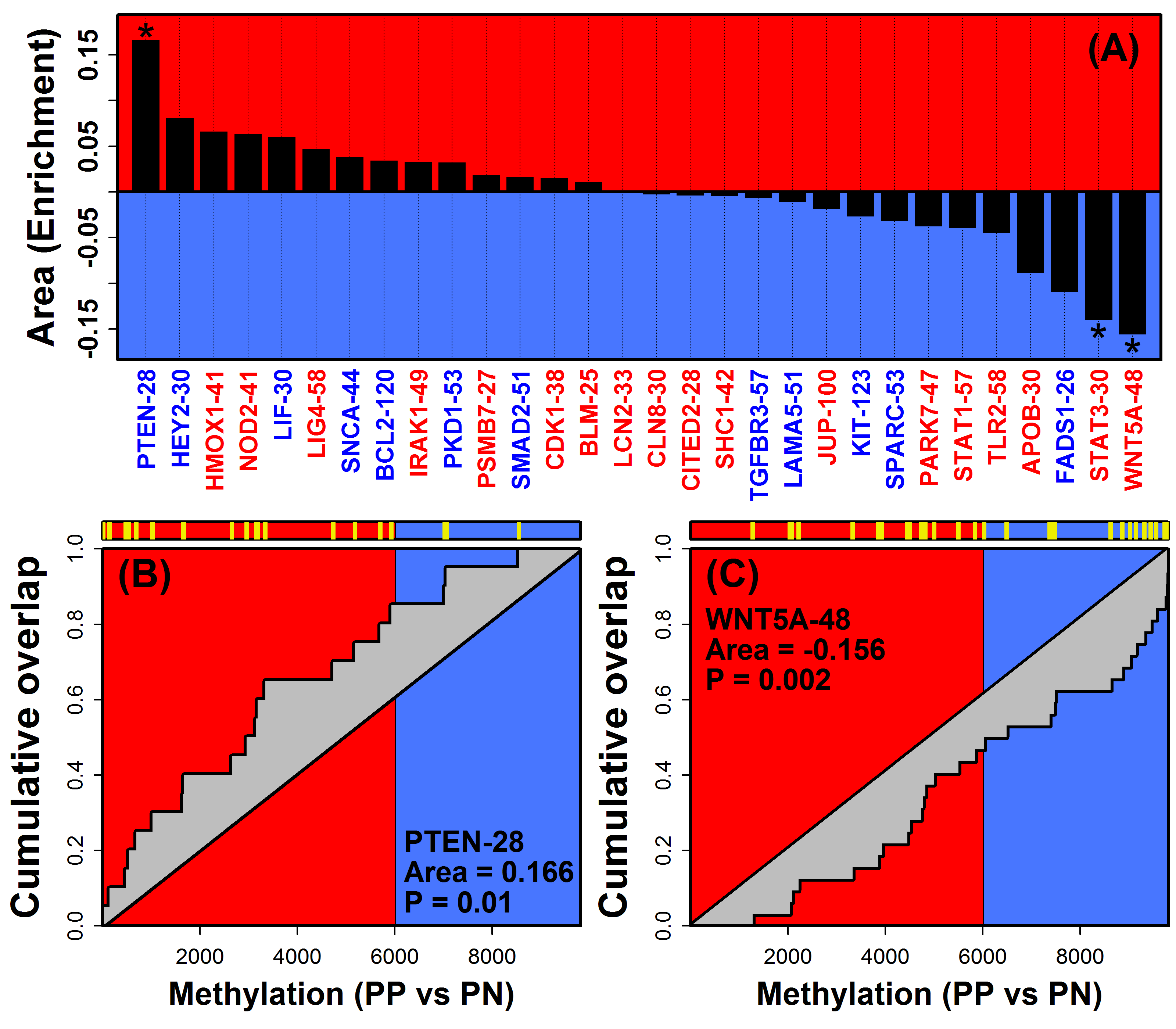

Supplement: Figure S12 — Identification of 3 DEMs biased towards hyper- or hypo-methylation in psoriasis lesions. The 30 DEMs were evaluated to determine if member genes were disproportionately hyper- or hypo-methylated in PP skin. (A) GSEA detection rate curve area statistics for each of the 30 DEMs (red labels, PP-increased DEMs; blue labels, PP-decreased DEMs). Significant statistics are denoted by an asterisk symbol (P<0.05, black asterisk). Parts (B) and (C) show GSEA results for PTEN-28 and WNT5A-48, respectively. For each human gene, the genomic site showing the strongest methylation difference between PP and PN skin was identified (i.e., lowest p-value). Human genes were then ranked according to the PP versus PN methylation difference estimated at this site (horizontal axis). Low ranks were assigned to genes hyper-methylated in PP skin (left, red region), while high ranks were assigned to genes hypo-methylated in PP skin (right, blue region). Yellow hash marks (top) denote placement of DEM genes with respect to each ranking, and the curve in each figure tracks the cumulative overlap of DEM genes from left to right (vertical axis). Enrichment of DEM genes among genes hyper-methylated in PP skin is indicated by a cumulative overlap curve above the diagonal (i.e., positive area statistic; Figure B). Enrichment of DEM genes among genes hypo-methylated in PP skin is indicated by a cumulative overlap curve below the diagonal (i.e., negative area statistic; Figure C). (TIF) [file pone.0079253.s012.tif]

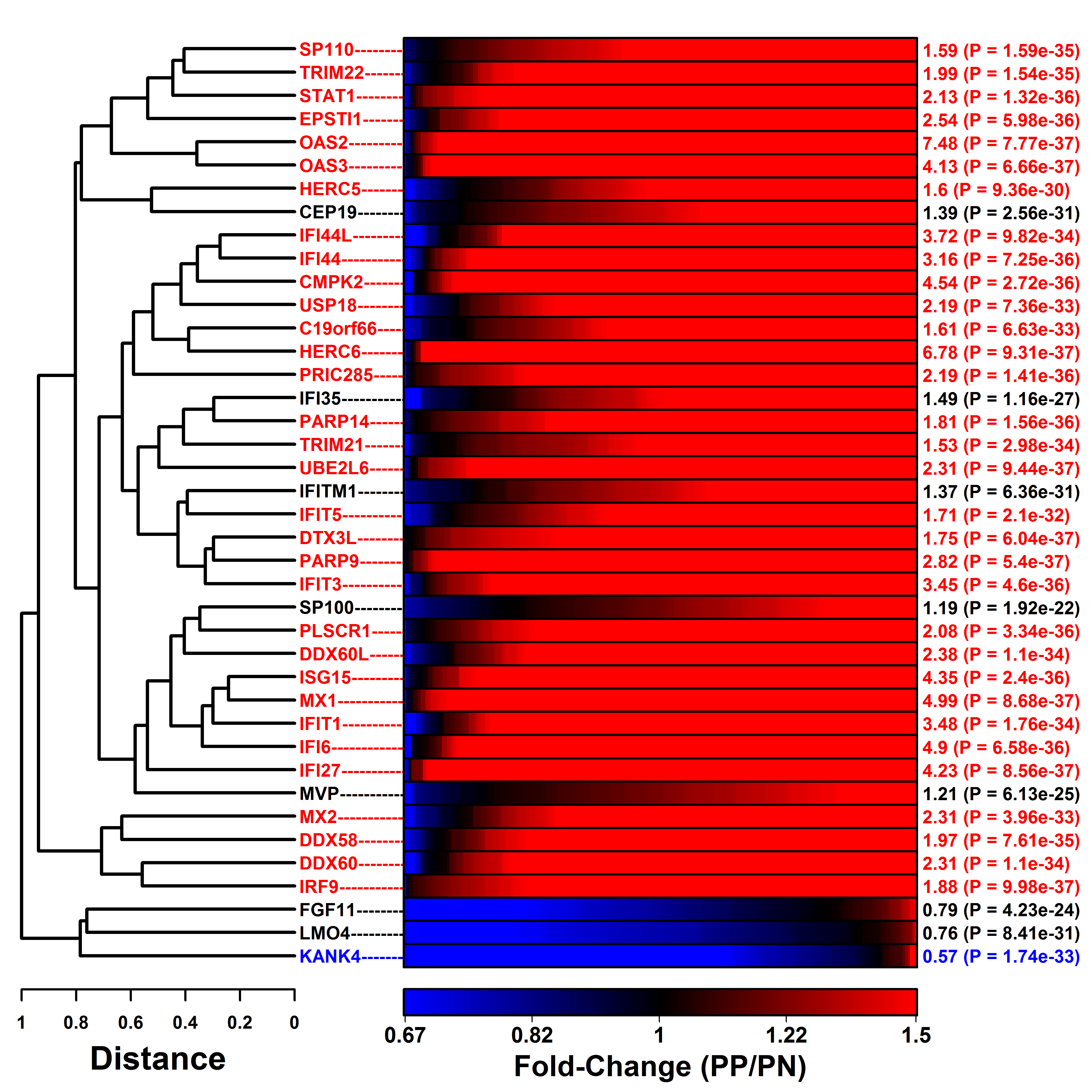

Supplement: Figure S13 — Expression of STAT1-57 genes in lesional (PP) versus uninvolved (PN) skin from psoriasis patients ( n = 215). The figure shows 40 genes from STAT1-57 with the most significant expression difference in PP versus PN skin (i.e., lowest p-value). Genes were clustered based upon their expression pattern in KC and epidermis microarray samples (Euclidean distance). For each gene, fold-change estimates (PP/PN) among the 215 patients were sorted and displayed in the figure (see color scale). The right margin lists the median fold-change with p-value from a non-parametric test for differential expression between PP and PN skin (Wilcoxon rank sum test). (TIF) [file pone.0079253.s013.tif]

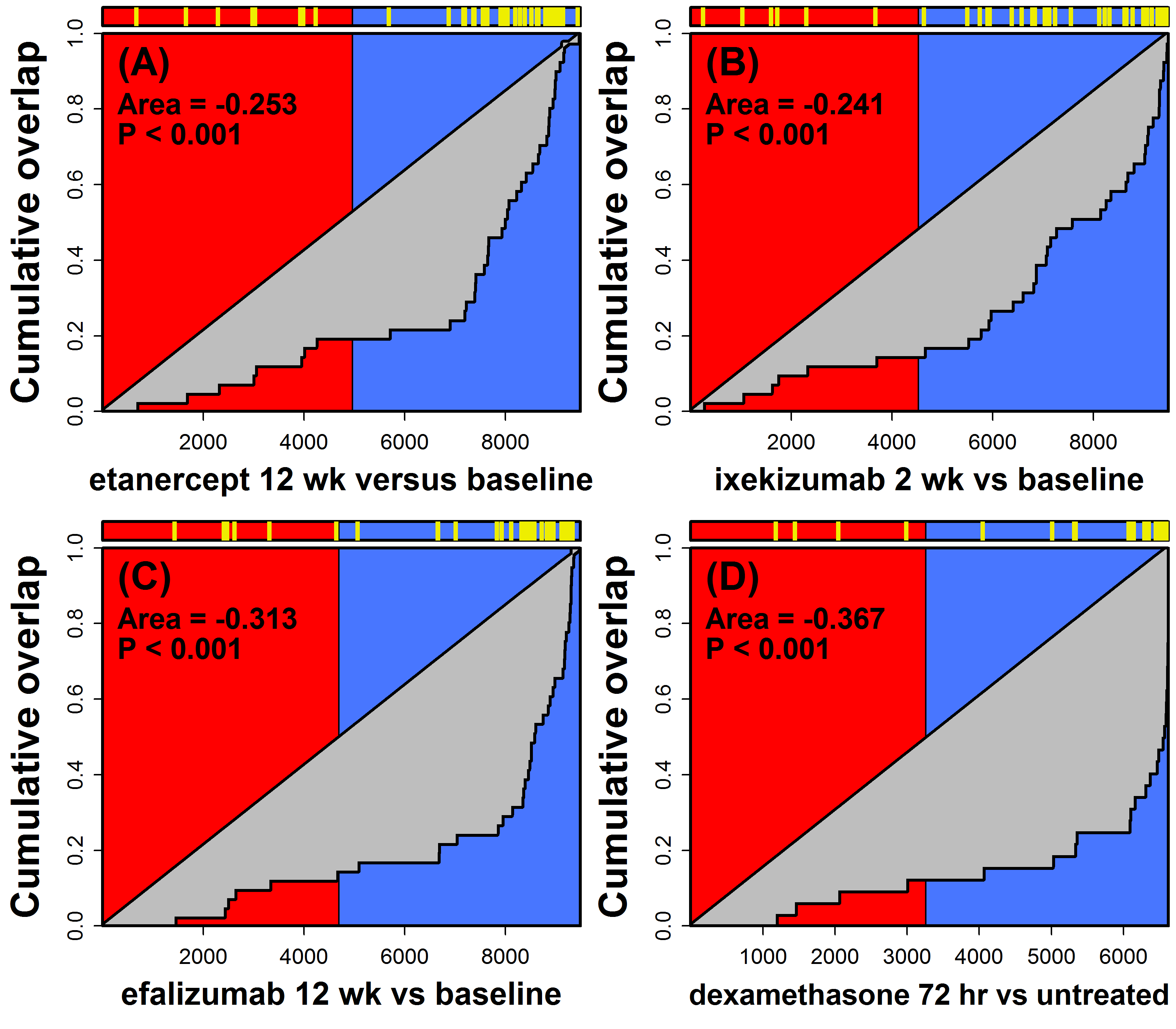

Supplement: Figure S14 — STAT1-57 is a glucocorticoid-inhibited DEM repressed in lesions following biologic therapy (etanercept, ixekizumab and efalizumab). Figures (A) – (D) show GSEA results evaluating whether the STAT1-57 genes are disproportionately increased or decreased in (A) PP lesions following 12 weeks of etanercept treatment versus PP lesions at baseline (GSE11903), (B) PP lesions following 2 weeks of ixekizumab treatment versus PP lesions at baseline (GSE31652), (C) PP lesions following 12 weeks of efalizumab treatment versus PP lesions at baseline (GSE30768) or (D) KCs treated with glucocorticoid (dexamethasone) versus untreated KCs (GSE26487). In each figure (A – D), genes were first ranked according to how their expression is altered in the indicated comparison (horizontal label). Red background denotes genes increased in each comparison while blue background denotes genes decreased in each comparison. Yellow hash marks (top) denote placement of the STAT1-57 genes with respect to each ranking, and the curve in each figure tracks the cumulative overlap of STAT1-57 genes with top-ranked genes from left to right (vertical axis). Enrichment of STAT1-57 members among genes decreased in each comparison is indicated by a cumulative overlap curve below the diagonal (i.e., negative area; Figures A – D). (TIF) [file pone.0079253.s014.tif]

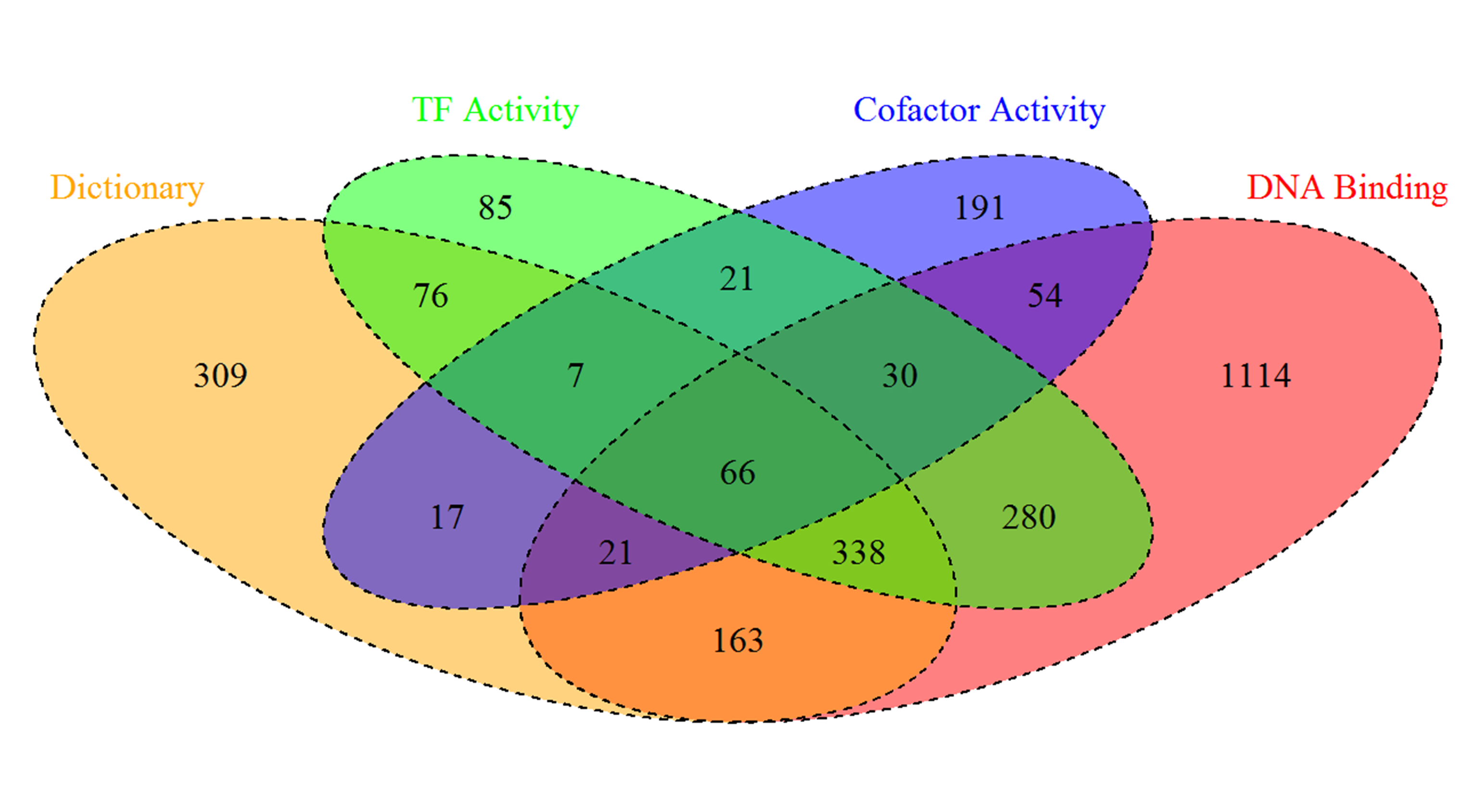

Supplement: Figure S16 — Gene ontology (GO) biological process (BP) terms and the 997 human genes associated with DNA motifs included within our dictionary. The 1937 DNA-binding motifs included within our dictionary were associated with 997 unique human genes. The Venn diagram shows the intersection of these genes with the complete set of human genes annotated with the GO BP terms “Transcription factor activity” (GO:0003700), “DNA binding” (GO:0003677), and “Transcription cofactor activity” (GO:0003712). (TIF) [file pone.0079253.s016.tif]
