# Supplementary material for: Modulation of Epidermal Transcription Circuits in Psoriasis: New Links between Inflammation and Hyperproliferation
Source: PLoS One. 2013 Nov 15;8(11):e79253. doi: 10.1371/journal.pone.0079253 (PMC3829857; doi:10.1371/journal.pone.0079253)
Supplement: Figure S15 — PRISMA flowchart for meta-analysis of psoriasis gene expression data. We followed Preferred Reporting Items for Systematic Reviews and Meta-Analyses (PRISMA) guidelines to evaluate gene expression in lesional (PP) and uninvolved (PN) skin from 215 psoriasis patients. The PRISMA flowchart provides an overview of the identification and screening steps followed to generate data from the 215 patients. In brief, we analyzed all studies that utilized the Affymetrix Human Genome U133 Plus 2.0 microarray platform (GEO accessions: GSE13355, GSE14905, GSE30999, GSE34248, GSE41662 and GSE41663), but excluded four studies in which less comprehensive microarray platforms had been utilized (GEO accessions: GSE2737, GSE6710, GSE11903 and GSE26866). (PDF) [file pone.0079253.s015.pdf]

PRISMA 2009 Flow Diagram

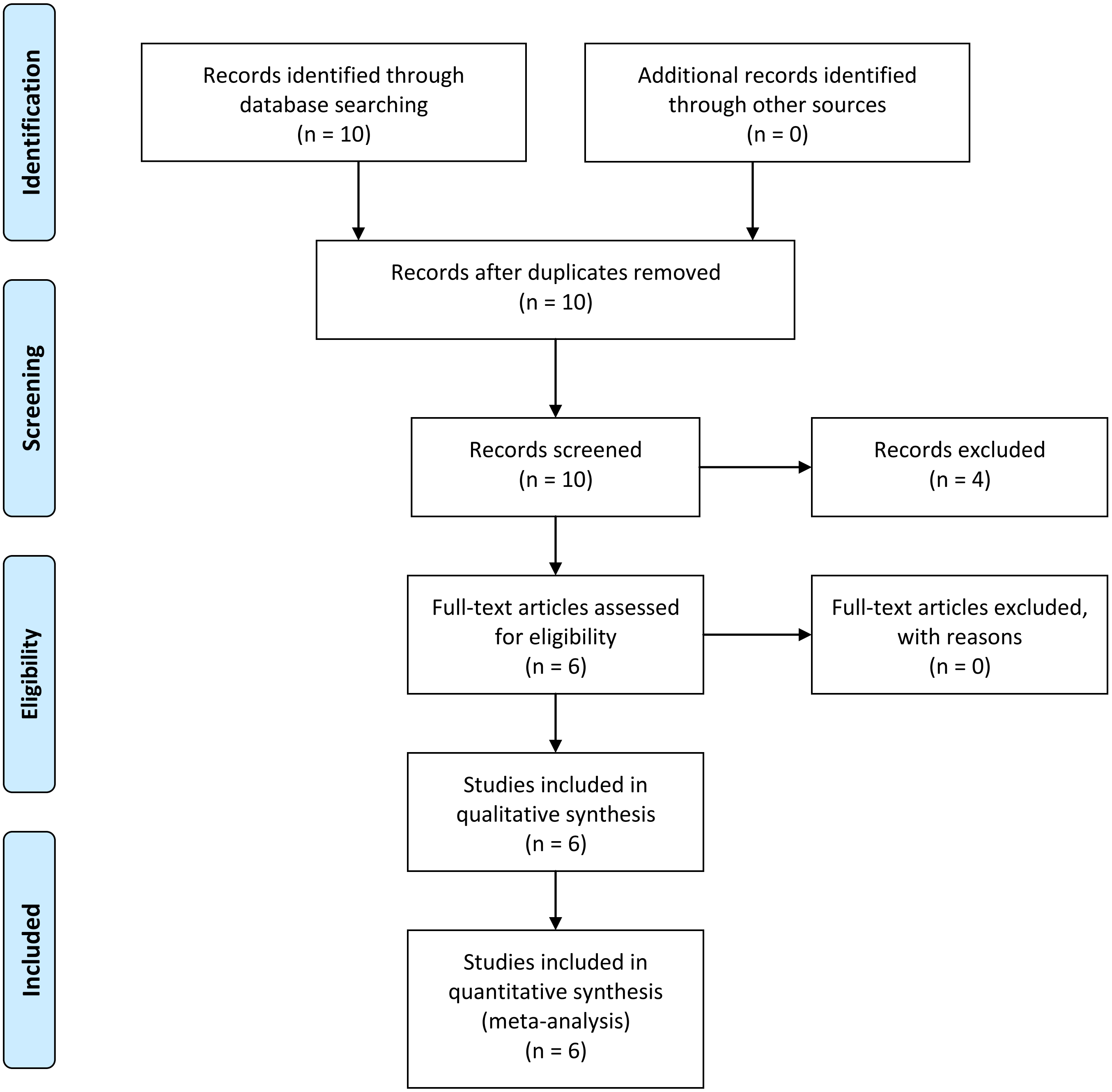

From: Moher D, Liberati A, Tetzlaff J, Altman DG, The PRISMA Group (2009). *P*referred *R*eporting *I*tems for *S*ystematic Reviews and *M*eta-*A*nalyses: The PRISMA Statement. PLoS Med 6(6): e1000097. doi:10.1371/journal.pmed1000097

For more information, visit www.prisma-statement.org.
