# Supplementary material for: Modulation of Epidermal Transcription Circuits in Psoriasis: New Links between Inflammation and Hyperproliferation
Source: PLoS One. 2013 Nov 15;8(11):e79253. doi: 10.1371/journal.pone.0079253 (PMC3829857; doi:10.1371/journal.pone.0079253)
Supplement: Table S1 — Description of cohorts used for microarray datasets ( n = 215 patients). The first column lists the Gene Expression Omnibus accession identifier under which raw data can be accessed. Label refers to the type of target nucleic acid (cRNA or cDNA) used for hybridization with the oligonucleotide array platform (Affymetrix Human Genome U133 Plus 2.0 array). Cohort characteristics are summarized in the final column based upon information provided in original research reports (see footnotes). (PDF) [file pone.0079253.s017.pdf]

Table S1. Description of cohorts used for microarray datasets (*n* = 215 patients). The first column lists the Gene Expression Omnibus accession identifier under which raw data can be accessed. Label refers to the type of target nucleic acid (cRNA or cDNA) used for hybridization with the oligonucleotide array platform (Affymetrix Human Genome U133 Plus 2.0 array). Cohort characteristics are summarized in the final column based upon information provided in original research reports (see footnotes).

Study	*n*	% male	Age	Type	Label	Cohort characteristics
GSE13355[a]	57	49.1%	mean: 48.9<br>range: 21 - 69	chronic plaque, mild to severe	cRNA	Psoriasis plaques not limited to the scalp area. Average total body surface area covered with lesions was 14.2%±1.7% (range: 1% – 62%). No systemic medications for 2 weeks prior to biopsies (1 week for topical).
GSE14905[b]	26	?	?	plaque type	cRNA	?
GSE30999[c]	80	77.5%	mean: 44.6	chronic plaque, moderate to severe	cRNA	Candidates for systemic treatment with > 10% body surface involved. No systemic agents used for 4 weeks prior to biopsy (2 weeks for topical). No biologic agent for 3 months prior to biopsy.
GSE34248[d]	14	71.4%	range: 23 - 71	plaque type, mild to moderate	cDNA	Patients reported no medication use to treat psoriasis.
GSE41662[d]	23	?	range: 19 - 55	plaque type, moderate to severe	cDNA	Clinically stable plaque psoriasis over ≥10% of body surface area and a Psoriasis Area and Severity Index score of ≥10.
GSE41663[d]	15	?	range: 18 - 70	plaque type, moderate to severe	cDNA	No topical or systemic therapy for 1 month before biopsies. Greater than 10% body surface area involvement. Patients were not experiencing flare at biopsy.

[a]Nair et al. 2009, Nat Genet 41:199-204
[b]Yao et al. 2008, PLoS ONE 3:e2737
[c]Suárez-Fariñas et al. 2012, J Invest Dermatol 132:2552-2564
[d]Bigler et al. 2013, PLoS ONE 8:e52242
